# Supplementary material for: A community-powered search of machine learning strategy space to find NMR property prediction models
Source: PLoS One. 2021 Jul 20;16(7):e0253612. doi: 10.1371/journal.pone.0253612 (PMC8291653; doi:10.1371/journal.pone.0253612)
Supplement: S1 Appendix — (PDF) [file pone.0253612.s001.pdf]

Supporting Information for A community-powered search of machine learning strategy space to find NMR property prediction models

S1 Overview

Section S2 and S3 contains details on how the dataset was generated, and what considerations went into the choices made. Section S4 describes the data set available to the partitipants, as well as details on how submissions were scored. Section S5 contains various plots and analysis strategies that didn't make it into the main paper, as well as details on how the ensembles were fitted and the correlations were calculated.
Section S7, S8, S9, S10, S11 and S12 contain detailed write-ups explaining the model architecture of the five winning teams as well as the 12th placed team.
Section S13 outlines the code and data available for download, including all the winning solutions. Section S14 contains the competition rules. And finally section S15 contains the description of the competition given to the participants.

S2 Dataset considerations

We opted to use structures from the QM9 dataset[1] to construct our own dataset as well as the same computational methodology. QM9 consists of around 130,000 molecules comprised of carbon, fluorine, nitrogen, oxygen and hydrogen. Each molecule has up to 9 heavy atoms (non-hydrogen) and up to 29 total number of atoms. Each structure was optimized by the authors using Density functional theory (DFT), with the B3LYP functional[2] and the 6-31g(2df,p) basis set[3, 4, 5, 6]. There were several advantages of basing our dataset on QM9, as well as several possible disadvantages that needed to be considered. QM9 has historically had an important role as a benchmark dataset in the development of machine learning models for 3D structure-property relationships, and we deemed it beneficial to augment this with magnetic properties. This is particularly true for pairwise properties like scalar coupling constants, as there to our knowledge currently does not exist any dataset that includes pair-wise properties. Using an existing set of structures also eased the workload of gener-

ating a dataset considerably. We initially had two primary concerns about using QM9 structures specifically and one primary concern about using computational methods in general.

- The QM9 molecules are quite small compared to molecules commonly used as e.g. medicines, and the solutions to the competition might not work (due to memory use etc.) on larger molecules. However, since the strength of atomic interactions decay with distance (with the exception of large aromatic systems), we believe that we could ultimately modify the solutions to scale better with system size by introducing an interaction distance cutoff. At this time, we have not researched the current size limit of the winning solutions.

- All of the QM9 molecules have been optimized to form a local minimum on the potential energy surface (equilibrium). In the future we might be interested in studying non-equilibrium structures, and the solutions might perform less well on these (such as methods ignoring 3D relationships, and that relies entirely on connectivity). All of the top solutions incorporated 3D relationships, and at this time we have no reason to believe that any of them will perform poorly on a non-equilibrium dataset.

- By relying on quantum chemistry calculations that directly relate a molecular 3D structure to the target property, participants could guess the specific methodology and compute the target properties of the test set, thus knowing the correct answer. Due to the computational program not being widely available, but more importantly due to the high computational cost (which directly translates to monetary cost), we expected that it was unlikely that anyone would 'cheat' in this way. This is particularly true as it was a requirement of the competition that the source code to the solution was made accessible to the organizers for a team to be eligible for any prize money.

S3 Dataset generation

Of the 130,831 molecules in QM9 that passed the geometry consistency check, we removed an additional 42 molecules as these had no hydrogen atoms. The remaining structures were modified to a valid xyz format and input files for Gaussian NMR computations were constructed. The scalar coupling constants (including contributions from separate terms), dipole moment vectors, nuclear shielding tensors, mulliken charges and potential energy were parsed from the Gaussian output files and the dataset was written in an easily accessible csv format. The molecule structures were made available in xyz formatted files as well as a single csv file. While the target observable was the scalar coupling constants, the auxiliary data was included in case additional learning from these could be achieved. A further 14 molecules were removed due to one or more of the scalar couplings being a big outlier compared to the range seen in the

remaining molecules. For a small subset of the remaining molecules (108) Gaussian automatically enabled symmetry in the DFT computations. This had little effect on most of the observables, however the direction of the dipole vector and the nuclear shielding tensor became arbitrary as a result of this (however the norm of the vector and trace of the matrix was still correct). This was discovered when the competition was ongoing, but since it only affected a small subset of the auxiliary data, the competition data was not updated with data computed with symmetry disabled.

S4 Competition details

The goal of the competition was to predict the scalar coupling constants from the interaction between a hydrogen and a hydrogen, carbon or nitrogen atom 1,2 or 3 bonds apart. This was to be done without the competitors knowing any information other than the coordinates and type of element of the atoms in the molecules. The dataset was randomly split into a 65%/35% training/test split. The scalar coupling distributions seemed to be very similar with random splits so stratified splits did not seem necessary.
The competitors were provided the following files:

- *structures.csv*, which contains the coordinates and element type of each of the 2,358,657 atoms of the 130,775 molecules that constitutes the combined test and training set.
- *train.csv*, which contains the values in Hz of the 4,658,147 scalar coupling constants in the training set as well as which atom pairs in which molecule the coupling is between.
- *test.csv*, which contains 2,505,542 pairs of atoms and their respective molecules, which the competitors had to predict the scalar coupling constants of.
- *potential_energy.csv*, which contains the potential energy in Hartree of the 85,003 molecules in the training set.
- *magnetic_shielding_tensors.csv*, which contains the XX, XY, XZ, YX, YY, YZ, ZX, ZY and ZZ components of the magnetic shielding tensors in ppm of the 1,533,537 atoms in the training set.
- *mulliken_charges.csv*, which contains the Mulliken charges in atomic units of the 1,533,537 atoms in the training set.
- *dipole_moment.csv*, which contains the dipole moments in Debye of the 85,003 molecules in the training set.
- *scalar_coupling_contributions.csv*, which contains the FC, SD, PSO and DSO components in Hz of the 4,658,147 scalar coupling constants in the training set. The sum of these equates to the values in *train.csv*.

The test set was further split into a 29%/71% public/private test set. All submissions were scored immediately on both splits. The public score was made available on the public leaderboard, while the private score (which the winners were determined from) were hidden until the end of the competition. here were no changes in the top 37 placements between the public and private leaderboard, indicating that there was little noise in the data.

We opted to use a custom score function to evaluate the submissions. The dataset included 8 different types of coupling: 1JHC (coupling between a hydrogen and a carbon separated by 1 covalent bond), 1JHN, 2JHH, 2JHC, 2JHN, 3JHH, 3JHC and 3JHN. Since the number of couplings of each type differed dramatically (811,999 3JHC couplings in the test set, but only 24,195 1JHN) and spanned different ranges, a bad choice of scoring metric could easily be dominated by the performance on the 3JHC coupling constants.

Our loss function is the logarithm of the geometric mean of the mean absolute error for each type:

$$\text{score} = \frac{1}{T}\sum_{t}^{T} \log\left(\frac{1}{n_t}\sum_{i}^{n_t} |y_i - \hat{y}_i|\right) \tag{S1}$$

Where:

- T is the number of scalar coupling types.
- n_t is the number of observations of type t.
- y_i is the actual scalar coupling constant for the observation i.
- $\hat{y}_i$ is the predicted scalar coupling constant for the observation i.

This way, a 10% improvement in one type of coupling will improve the score by the same amount as a 10% improvement in another type of coupling.

S5 Analysis

S5.1 Ensembling

We created an ensemble of the top 400 submissions to see which submissions contributed the most. Of the top 400 submissions, 18 were removed due to being duplicates. The data points (the test set referenced above) were split into a training and test set of equal size, stratified by coupling type using the Scikit-learn library[7]. We did a linear fit to the submissions using TensorFlow[8], minimizing the loss function in equation S1 under the constraint that the weights were positive and summed to 1. 6 submissions had weights of larger than 0.02 as shown in Table S1.

Similarly we did separate linear fits for each coupling type that minimized the mean absolute error. A comparison of the performance of the individual teams and the ensembling strategies described above is shown in Table S2

Table S1: Competition rank, team name and individual weight of 6 teams whose submission had a weight greater than 0.02 in the ensemble.

Rank	Name	Weight
1	hybrid	0.204
2	Quantum Uncertainty	0.270
3	[kakr] Solve chem together	0.111
4	Hyperspatial Engineers	0.203
5	DL guys	0.046
12	Team Bird Brain	0.149

Table S2: The performance of the ensemble fitted on all coupling types ('E'), the ensemble fitted on each coupling type separately ('E*'), and the submitted solutions (individual contributions and the total score per coupling type).

Rank	1JHC	1JHN	2JHH	2JHC	2JHN	3JHH	3JHC	3JHN	Total
E	-0.296	-0.311	-0.509	-0.432	-0.463	-0.502	-0.418	-0.483	-3.414
E*	-0.297	-0.332	-0.515	-0.433	-0.463	-0.507	-0.421	-0.485	-3.453
1	-0.284	-0.288	-0.477	-0.410	-0.440	-0.477	-0.400	-0.464	-3.241
2	-0.277	-0.291	-0.458	-0.410	-0.450	-0.474	-0.394	-0.472	-3.226
3	-0.269	-0.244	-0.480	-0.411	-0.443	-0.488	-0.397	-0.461	-3.193
4	-0.267	-0.286	-0.482	-0.401	-0.433	-0.470	-0.390	-0.455	-3.184
5	-0.275	-0.289	-0.468	-0.400	-0.428	-0.452	-0.387	-0.450	-3.149
12	-0.221	-0.278	-0.448	-0.357	-0.414	-0.444	-0.336	-0.437	-2.936

S5.2 Correlation

We investigated how similar the submissions from the top teams were by computing the correlation between a scaled subset of their submissions on the test data. 20,000 data points for each coupling type were drawn randomly from the test set. For each coupling type the submissions were the scaled by their individual root mean square error (RMSE). These two pre-processing steps were done to make each coupling type have an equal impact on the correlation.

The same analysis was done on the top 50 team submissions where the submissions were clustered with hierarchical clustering using the 'complete' linkage in the Scipy module[9].

S5.3 Manifold

In a similar analysis, we projected the top 100 submissions of a scaled subset of the test set down on a two-dimensional manifold. Again, 20,000 data points for each coupling type were drawn randomly from the test set. As the mean absolute error (MAE) is a more natural metric in relation to the scoring metric used in the competition, the submissions were for each coupling type scaled by their

individual MAE. This pre-processing is important as if the submissions weren't scaled appropriately, the dimensionality reduction might mainly capture the average performance of a given submission, rather than how the methods differ in general.
Figure S1 shows the submissions projected onto a two-dimensional Multidimensional Scaling (MDS) manifold (which tries to preserve the Manhattan distance between submissions)[7, 10, 11, 12].

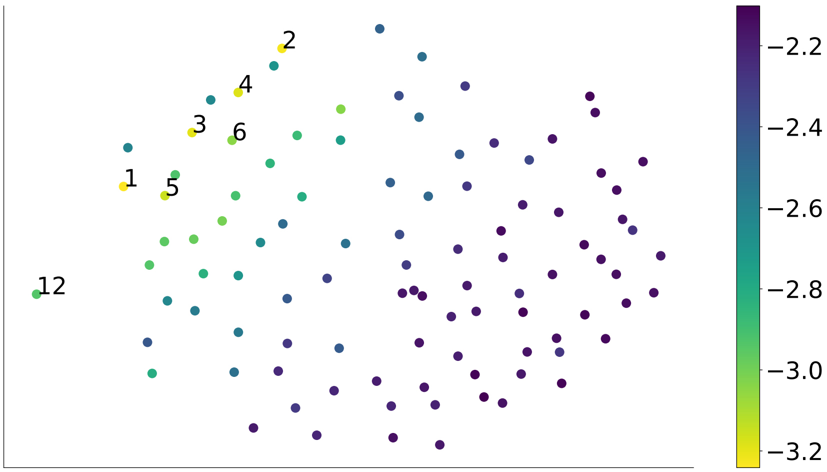

Figure S1: Projections of top 100 submissions to a two-dimensional MDS manifold. Coloring indicate submissions score as indicated by the colorbar. A subset of 7 submissions are marked with the competition rank.

S5.4 Comparison with previous competitions

To get an idea of how engaging the competition was to the community (and earlier how much participation we could expect), we looked at how the number of participating teams have historically depended on the prize pool. We retrieved the number of participating teams for all previous competitions with a monetary prize and converted the prize into US dollars. Additionally we restricted ourselves to competitions where everyone could enter, where the number of teams were listed, that were not of recruitment or code-type, and competitions that took place within the last five years. Figure S2 shows how this competition compare to previous ones.

S5.5 Participant engagement

We looked at how many days separated each participating teams' first and last submission, to get an overview over the average engagement. Figure S3 shows

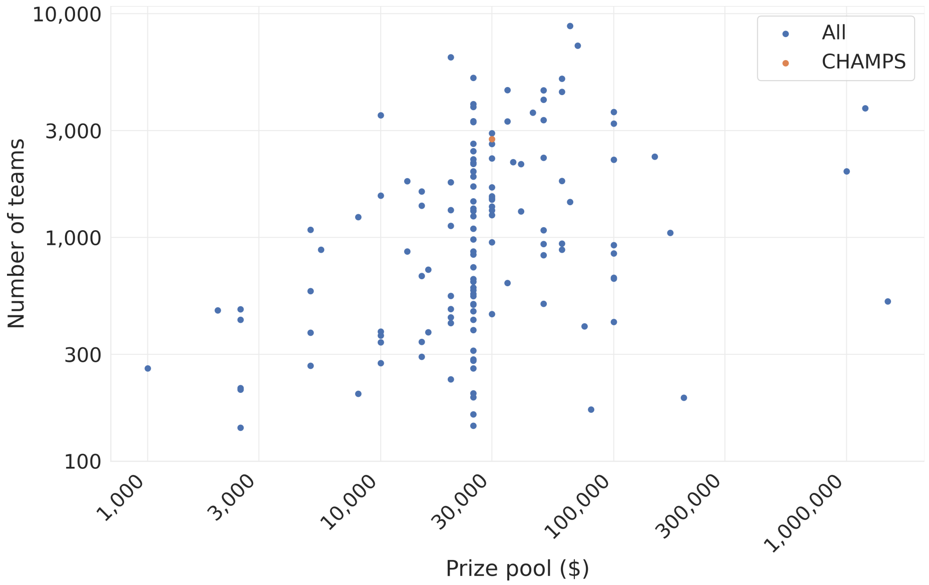

Figure S2: How the prize pool are related to number of participating teams on Kaggle in the last five years (log-log plot). This competition highlighted in orange.

that many teams concentrated their efforts (or did only a single submission) over 1-2 days. However, Figure S4 truncates any team that made continuous submissions over a period longer than 3 weeks into a single bin, showing that the majority of the teams were engaged throughout the competition

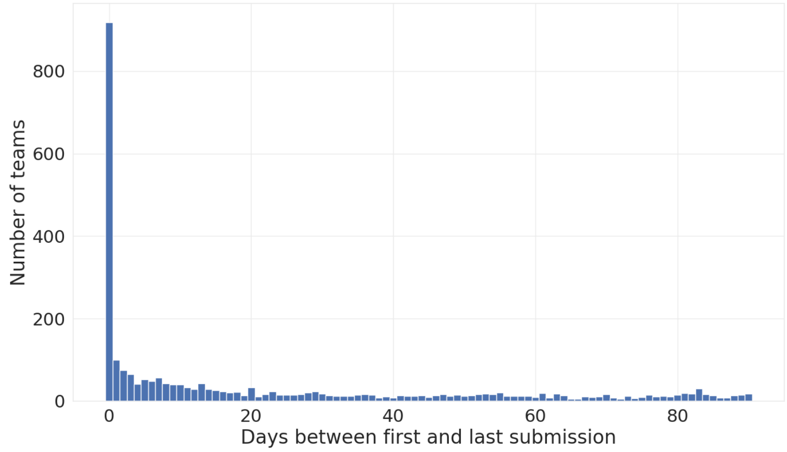

Figure S3: Histogram showing the number of days between a teams' first and last submission.

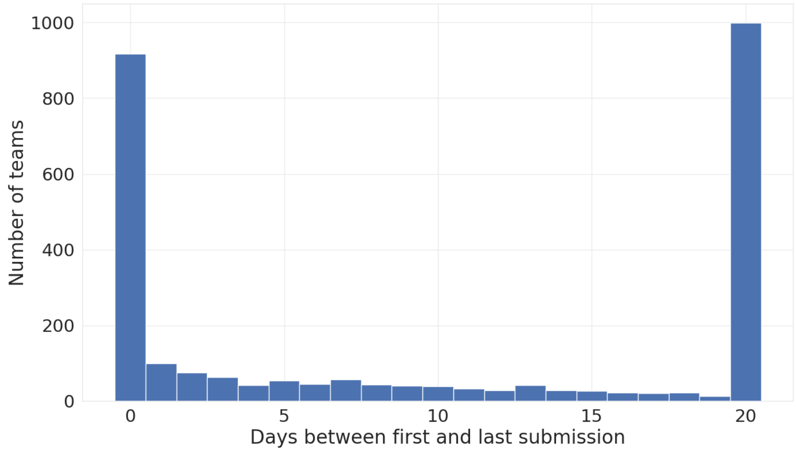

Figure S4: Histogram showing the number of days between a teams' first and last submission, where the rightmost bin indicate teams with more than 3 weeks between first and last submissions.

S6 Comparison to IMPRESSION

In the manuscript we provide comparisons to our previously published methodology (IMPRESSION [13]). We report ratio's in a range, where the upper bound are the best models that we have trained on the training set. These could likely be improved upon using higher memory machines.
As the error in kernel methods tend to scale as a power law with number of data points, we extrapolated what the error theoretically could be in the limit of using the entire training data set. This estimate is used as the lower bound. Table S3 shows the score contribution of each coupling type for the best ensemble, the winning submission as well as scores for IMPRESSION best trained models and interpolated errors. Similarly Table S4 shows the mean absolute errors.

Table S3: Score contributions for each coupling type for the best ensemble, the winner and IMPRESSION lower and upper bounds. (lower, upper)

Type	Ensemble*	Winner	IMPRESSION
1JHC	-0.296	-0.284	(-0.150, -0.003)
1JHN	-0.332	-0.288	(-0.256, -0.178)
2JHH	-0.515	-0.477	(-0.209, -0.119)
2JHC	-0.433	-0.410	(-0.238, -0.018)
2JHN	-0.463	-0.440	(-0.283, -0.143)
3JHH	-0.507	-0.477	(-0.048, 0.019)
3JHC	-0.421	-0.400	(-0.123, 0.049)
3JHN	-0.485	-0.464	(-0.216, -0.107)
Sum	-3.453	-3.241	(-1.522, -0.499)

Table S4: Mean absolute error for each coupling type for the best ensemble, the winner and IMPRESSION lower and upper bounds.

Type	Ensemble*	Winner	IMPRESSION
1JHC	0.0937	0.1031	(0.3002, 0.9728)
1JHN	0.0702	0.0999	(0.1292, 0.2404)
2JHH	0.0162	0.0220	(0.1886, 0.3872)
2JHC	0.0313	0.0376	(0.1488, 0.8682)
2JHN	0.0246	0.0296	(0.1040, 0.3180)
3JHH	0.0173	0.0220	(0.6815, 1.1675)
3JHC	0.0345	0.0408	(0.3751, 1.4811)
3JHN	0.0207	0.0244	(0.1780, 0.4257)
Geometric mean	0.0317	0.0391	(0.2183, 0.6069)

S7 Solution 1 - Hybrid

S7.1 Features

Of the provided data, we use only the element and position of each atom and the scalar coupling type of each bond. From these, we use the open source package RDKit[14] to generate additional features for each atom, specifically bond order and partial charge. Below we list the total set of features of each molecular object that we use in the network.

- Atoms: element, number of neighbors, order of neighboring bonds, partial charge, angle with nearest neighbors. All atoms are included.
- Bonds: each atom's element, coupling type (if applicable), bond order (if applicable), distance. These are included regardless of whether there is a chemical bond or not.
- Triplets: each atom's element, bond angle. These are included only if there is a chemical bond (one central atom bonded to a pair of other atoms).

The methods described can be generalized to include quadruplets (and dihedral angles) as well, although the addition of quadruplets was found to not be helpful in this problem.

S7.1.1 Molecular Representation

Deep learning approaches to problems in molecular modeling generally represent the molecule as a graph, with nodes representing atoms and edges representing bonds. Many of these are then passed through networks that can be described using the general framework of message passing neural networks (MPNN)[15], where information is usually passed through alternating convolutions, first from edges to nodes and then from nodes to edges. This representation is restrictive for several reasons. Most importantly, information may only be passed locally, as direct connections do not exist between, for example, pairs of atoms, or an atom and a bond far away in the molecule. Further, this molecular representation only allows us to use features that directly correspond to a particular atom or bond; for example, it is not clear how to incorporate features of atom triplets as input in a typical MPNN. In contrast to this framework, we represent each molecular object of interest (in this case, each atom, bond, and triplet, but we may incorporate other objects or properties of the molecule as we like) as a node in a complete graph.

S7.2 Model Architecture

Our architecture is a deep learning approach with three stages: an embedding layer, several graph transformer layers, and element-wise group convolutions. Each of these is detailed in the subsections below.

S7.2.1 Embedding

We use three different embedding layers, each corresponding to a type of molecular object (atom, bond, and triplet). All of these objects have a mixture of discrete and continuous features, and some of these features are hierarchical in nature (for example, bonds' coupling type and sub-type, which includes some local bond-order information). As such, we use hierarchical embeddings[16], which embed each feature separately, and then linear combine these embeddings to yield a single representation of the object. Discrete features are embedded in the usual fashion, while for continuous features we use a sine filter embedding similar to the position embeddings employed by Transformer models[17]. We embed all features described in Section S7.1, with the notable exception of atom positions. Instead, we use these positions to construct a matrix of relative distances to be used later in the network; this ensures that network output is invariant to translation and rotation of the molecule.

Subsequent layers in the network will convolve over all nodes in the graph; as such, we require that the output size of each of these embeddings be the same. It is worthwhile to note that after the embedding layer, all nodes are treated identically by the network – that is, the graph transformer layer makes no distinction between nodes represented by atoms, bonds, and triplets. Rather, all representation of molecular objects exist in the same space, and it is the job of the embedding layer parameters to learn representations that can be meaningfully distinguished by the remainder of the network.

S7.2.2 Graph Transformer Layers

Throughout this section, we let Z be a matrix that represents the hidden layer of a molecule, where each column of Z represents a particular node. The layer describe here, which we call the graph transformer layer, forms the foundational building block of our architecture. This layer is an extension of a graph convolution layer, which, in its most general form, may be represented as

$$\mathbf{GraphConvolution}(Z) = \sigma(WZA) \tag{S2}$$

where σ is an activation function, W is a matrix of learnable weights, and A is a static mixing matrix that encodes the structure of the graph; common choices for A include a normalized adjacency matrix or a graph Laplacian. Our first modification is the replacement of the mixing matrix A with a self-attention mechanism, which was popularized by the Transformer model[17] for sequence modeling. In self-attention, the strength of message passing is computed via a parametrized inner product between nodes:

$$\mathbf{GraphSelfAttention} = \sigma(W_3 Z \quad \mathbf{softmax}(Z^T W_1^T W_2 Z)) \tag{S3}$$

Under this architecture, W3 learns the message to be passed, while the weight matrices W1 and W2 learn the strength of each message. Replacing the mixing matrix A in this manner removes the only structural information the network

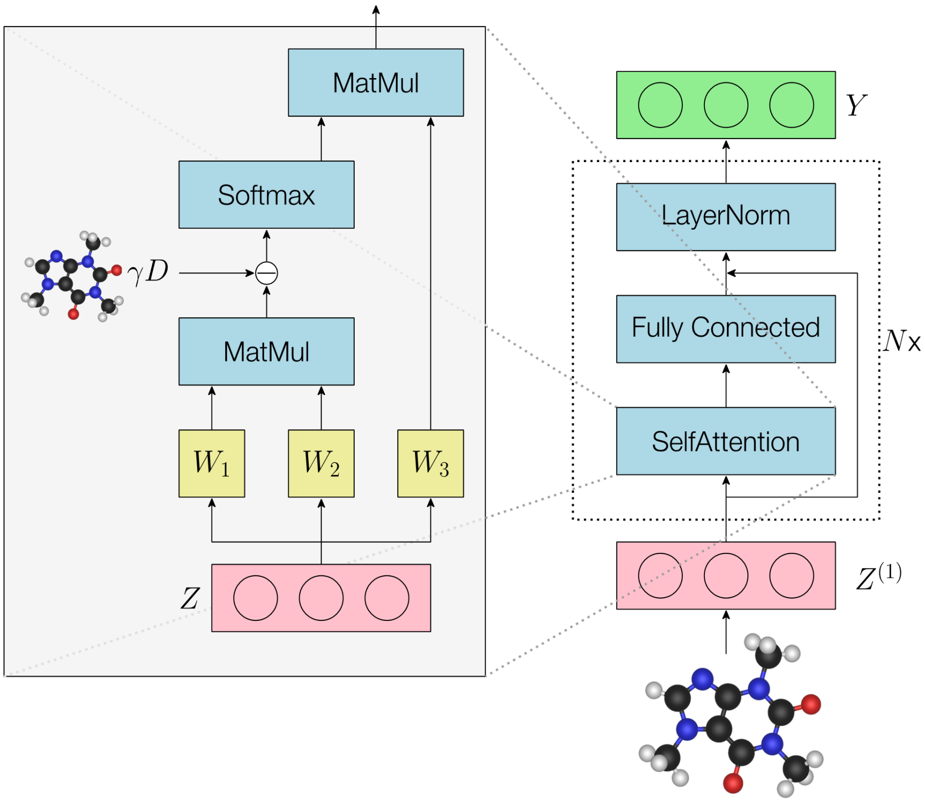

Figure S5: Graphical representation of the model architecture.

receives about the graph. To reintroduce this information, we incorporate a scaling based on the distance between each pair of nodes in the graph. Let D be a matrix such that $D_{i,j}$ represents distance between nodes i and j, and let γ be a learnable scalar parameter. Distance-scaled self- attention is given by

$$\textbf{ScaledGraphAttention}(Z) = \sigma(W_3 Z \quad \textbf{softmax}(Z^T W_1^T W_2 Z - \gamma D)) \quad \text{(S4)}$$

This scaling has the effect of reducing the strength of interaction between pairs of faraway nodes. The learnable parameter γ allows us to learn the intensity of this scaling: as γ approaches 0, relative distances are ignored, and as γ approaches ∞, we operate on a sparse graph, with information only flowing between directly adjacent molecular objects. The scaled graph self-attention transformation is shown in Figure S5. As in the original Transformer model, we follow self-attention with a linear transformation, a residual connection, and

layer normalization[18]. The full graph Transformer layer is given by

$$\text{GraphTransformerLayer}(Z) \tag{S5}$$
$$= \text{LayerNorm}(W_4 \text{ScaledGraphSelfAttention}(Z) + Z) \tag{S6}$$
$$= \text{LayerNorm}(W_4 \sigma(W_3 Z \text{softmax}(Z^T W_1^T W_2 Z - \gamma D)) + Z) \tag{S7}$$

S7.2.3 Defining Distances

The self-attention scaling described above requires a matrix D of relative distances between objects represented in the graph. However, since bonds and triplets do not have a well-defined position in space, it is not necessarily trivial to define distances among these objects. To do so, we begin with distances between two atoms a and a', denoted $d_{atom}(a, a')$, which we define as simple Euclidean distance between their positions. Distance involving larger sets of atoms are defined recursively, and with the guiding principle that the distance between two sets of atoms should be 0 if and only if one set is contained in the other. Distances are defined as below. In the following, let a be an atom, B be a bond connecting atoms b1 and b2, and C be a triplet containing center atom c1, other atoms c2 and c3, and bonds c12 and c13 .

$$d_{\text{atom,bond}}(a, B) = \min(d_{\text{atom}}(a, b1), d_{\text{atom}}(a, b2)) \tag{S8}$$
$$d_{\text{bond,bond}}(B, B') = \frac{1}{4} \sum_{b \in B, b' \in B'} d_{\text{atom,bond}}(b, b') \tag{S9}$$
$$d_{\text{atom,trip}}(a, C) = \min_{c \in c_1, c_2, c_3}(d_{\text{atom,atom}}(a, c)) + d_{\text{atom,atom}}(a, c_1) \tag{S10}$$
$$d_{\text{bond,trip}}(B, C) = \min(d_{\text{bond,bond}}(B, c_{12}), d_{\text{bond,bond}}(B, c_{12})) \tag{S11}$$
$$d_{\text{trip,trip}}(C, C') = \frac{1}{4} \sum_{c \in c_{12}, c_{13}, c' \in c'_{12}, c'_{13}} d_{\text{bond,bond}}(c, c') \tag{S12}$$

S7.2.4 Group Convolutions

After being passed through some number of graph transformer layers, we take the nodes representing coupling bonds and pass them through a series of 1×1 convolutions inside of a residual block. These convolutions are grouped according to coupling type and bond order, such that the set of convolutional filters applied is different for each group. A final grouped convolution outputs the predicted scalar coupling constant.

S7.3 Training and Ensembling

In total, 13 of the models described in the previous section were trained on the task of predicting magnetic scalar coupling constants. Model size varied, but

generally had between 12 and 18 graph transformer layers, and hidden dimension between 600 and 800. These models were initially trained using absolute loss instead of the loss defined in equation S1. The model was implemented in PyTorch[19] and network training was done using the ADAM optimizer[20] with a cosine annealing learning rate scheduler[21] for 200 epochs. For some of our best models, we additionally fine-tuned by training for approximately 40 epochs using the loss defined in equation S1 to improve their single model scores by around -0.020 to -0.030. For each coupling subtype (i.e. coupling type, plus further breakdowns by element and chemical environment information), the targets were scaled to 0 mean and one standard deviation to simplify the learning process. These 13 models were ensembled with a mix of mean and median ensembling. First, for each coupling type, we observed how frequently each model's prediction was the median among all 13 predictions. The 9 best models according to this metric were selected, with the remaining 4 discarded. Finally, for each coupling constant, we selected the center 5 median values from among the 9 model predictions; the mean of these 5 values was used as the final prediction. We found that this ensembling strategy was more effective than strict mean or median ensembling, and did not require a validation set to estimate individual models' test accuracy.

S8 Solution 2 - Quantum Uncertainty

S8.1 Data Augmentation

The key to the success of this model lies in the data augmentation, and comes from the insight that pair-wise properties can be modelled as atom-wise properties, given that relative position to the paired atom is encoded in the input. This is achieved by making duplicate 'sibling' molecules that are translated to have a different atom as origin.

For a given molecule of N atoms, where M of these are of atom type H, C or N, M siblings are constructed, each centered on a different H, C or N atom. Furthermore the sibling molecules are padded with dummy atoms to make all molecules have the same number of atoms (29 for this competition), which are later masked in the model. Each atom in the sibling molecules are then matched with the corresponding coupling constant label, where dummy values are used for coupling types that are not present in the dataset, and these are similarly masked later in the model.

S8.2 Input Features

A feature vector is constructed for each atom in the sibling molecules, that contain the Cartesian coordinate of the atom, the atom type index (C=0, H=1, ...) as well as an index for the type of coupling formed with the origin atom (1JCH=0, 2JHH=1, ...). In the feature vector 3 elements will be the X, Y and Z component of the Cartesian coordinate, while the remaining elements are used

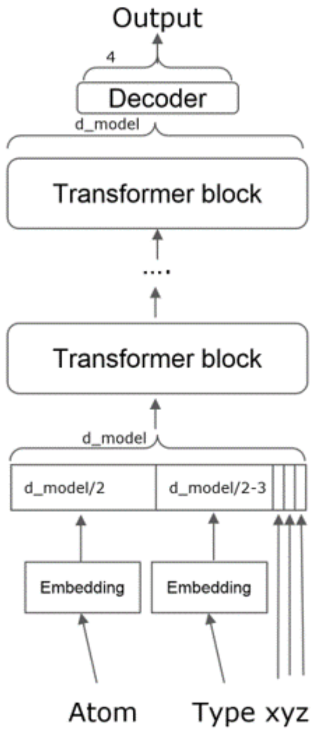

Figure S6: Overview of the overall architecture

for embedding the atom type and coupling type. For a feature vector of e.g. length 1024, 510 elements will be repetitions of the atom type index, 511 will be repetitions of the coupling type index, while the remaining 3 elements will contain the Cartesian coordinates. Note that there is no graph information nor any other manually engineered features.

S8.3 Model Architecture

The model used a standard transformer architecture [22] utilizing the fast.ai library [23], where each feature vector are updated with several transformer layers followed by a feed forward neural network to decode the scalar coupling constants from the transformed feature vectors (See Fig S6). Adding rotations during training didn't improve the model performance, indicating that the molecules were either systematically aligned in a way that enabled efficient training, or that rotation invariance were easily learned by the model.

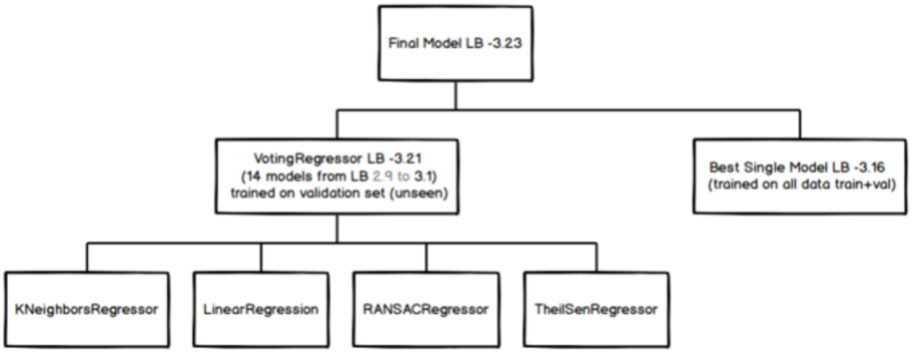

Figure S7: Overview of the ensembling strategy

S8.4 Training and Ensembling

14 models were trained, with varying dimensions from 512 to 2048 and layers from 6 to 24, and with scores of -2.9 to -3.1 Each model parameter size ranged from 12M to 100M (biggest model). A small validation set were kept separate to fit the ensemble model. Four different regressors were trained on the validation set predictions of the 14 models: k-nearest neighbors, linear, RANSAC [24] and Theil-Sen [25] regression models. A voting regressor were using to combine predictions of the four models, which resulted in a score of -3.21. Finally the single model with the best score (6 layers, 1024 dimensional feature vector) were trained on all the training data (including validation set), yielding a score of -3.16. Averaging this best single model with the voting regressor model yielded a final score of -3.23 on the public testing set. An overview of the ensembling strategy is shown in Fig S7. Predictions from the single best model takes ∼10 minutes for the test set of 46K molecules (∼15 ms per molecule).

S9 Solution 3

S9.1 Features

Due to the graphical nature of molecular systems, graph-based architectures are often applied to many chemical problems including this competition [26]. However, the nature of problem in this competition is slightly different to typical chemical problems, since atom-pair properties of three-dimensional chemical structures has not been modelled before and only a subset of atomic pairs in the molecules have target values (coupling constants). For example, the largest molecule in the training set (nonane) has 29 atoms and there are 406 atomic pairs. Among those atomic pairs, only 127 atomic pairs have target values. For this reason, an alternative representation for molecular systems than the graph representation can be utilized. Here, we use a set of only a part of pair features as a descriptor for a molecule. Even though we replace the graphical

representation, the size-extensiveness and permutation-invariant nature should be preserved. A similarity-based attention (or simply attention) is one of the mechanisms that satisfies both conditions:

$$\text{Attention}(Q, K, V) = \text{softmax}(\frac{QK^T}{\sqrt{d_k}})V \tag{S13}$$

where Q,K,V and d_k are query, key, value, and feature dimension of the key value respectively. An output of attention is a weighted average of input values where the weights are determined by similarity of queries and keys. Attention layers hold permutational invariance and size-extensivity because if the size or order of the input vector is changed, those of corresponding key and query vectors are changed. In many different contexts of machine learning, a large number of variants of attention have been proposed. A multi-head attention is one of the most frequently employed ones. It generalizes conventional attentions with concatenating results of attention whose inputs are linearly transformed with different weights.

$$\text{Multihead}(Q, K, V) = \text{Concat}(O_1, O_2, ..., O_h)W^0 \tag{S14}$$

$$O_i = \text{Attention}(QW_i^Q, KW_i^K, VW_i^V) \tag{S15}$$

where W_i^Q, W_i^K, W_i^V and W^O are weights for query, key, value, and outputs of attentions respectively. One of the benefits of using (multi-head) attention is to minimize sequential computing which is an obstacle for parallelization.

S9.2 Model Architecture

Transformers, one of the well-known natural language processing architectures, recorded overwhelming performance in the Seq2Seq[27] problem with replacing sequential computing to multi-head attention. The original transformer model[22] includes positional embedding to impose sequential information on input and output feature vectors before performing the encoding and decoding process, since encoder and decoder are composed of permutation-invariant layers. Also, like ordinary Attention and Linear blocks, they are size extensive which means different lengths of sequence can be treated. Hence, we employ encoder of Transformer to embedding pair sequence information to construct the latent space that contains interaction among atomic pairs. In terms of NLP, atomic pair information and pair sequences become words and sentences respectively. We refer to the original transformer paper for more details on the attention and transformer architecture [22]. Once again important features of the model is size-extensivity and permutation-invariance.

Our model (shown in Figure S9) adopts the encoder of Transformer architecture because of its size extensivity and permutation invariance. We make an atomic pair sequence to represent a molecule and calculate a target value by considering interactions among atomic pairs. The Transformer encoder (grey part in Figure S9) is employed to transfer input feature to latent space which is

Encoding	Number of encoder layers<br>Number of heads for attention<br>Dropout Ratio<br>Intermediate size	8<br>8<br>0.1<br>2048
Readout	Dimension<br>Dropout Ratio	832<br>0.1
Embedding	Embedding Dimension for Atomic Charge<br>Embedding Dimension for Position<br>Embedding Dimension for Atomic Number<br>Embedding Dimension for Distance<br>Embedding Dimension for Type	32<br>256<br>64<br>64<br>64
Data Augmentation	Mean of translational noise (Angstrom)<br>Standard deviation of translational noise (Angstrom)<br>Mean of angle in rotational noise (rad)<br>Standard deviation of angle in rotational noise (rad)	0<br>2<br>0<br>1.57
Dummy Types	1JHO, 1JCO, 1JCN, 1JNO, 1JCC, 1JNN, 1JFC	
Training and Ensemble	Learning rate<br>Linear warmup step<br>Weight Decay<br>Size of seed ensemble<br>Mean of initial weights distribution<br>Standard deviation of initial weights distribution	0.0003<br>30<br>0.01<br>10<br>0<br>0.02

Figure S8: Detailed solution information

read by Readout (purple part in Figure S9, more detailed information in Figure S10). For each type, weights of Readout are different but structures and dimensions of weights are the same. (see Table S8)

S9.3 Readout stage

Readout stage evaluates the spin coupling constant (SC in Figure S9) from the output sequence, output of the encoding layer. Here, we employ physical condition; the spin coupling constant is the sum of 4 components, Fermi Contact contribution (FC), Spin-dipolar contribution (SD), Paramagnetic spin-orbit contribution (PSO) and Diamagnetic spin-orbit contribution (DSO). The Readout stage is designed to predict a target value as mean of two differently evaluated values. One directly comes from the latent space and the other one is a sum of 4 components which comes from latent space. The loss function for the whole

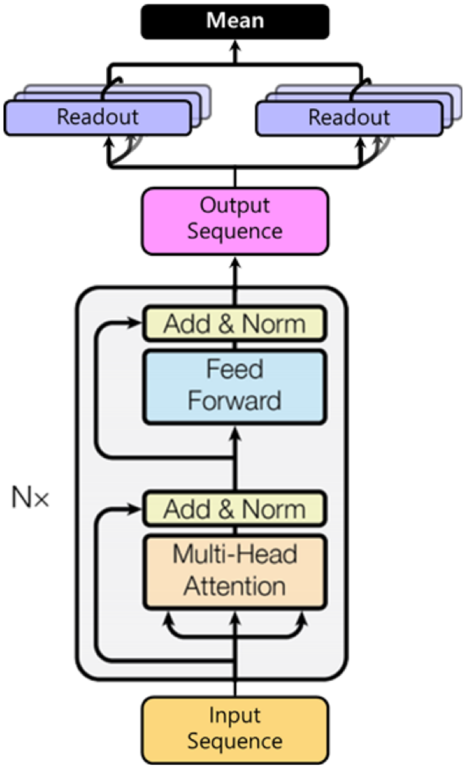

Figure S9: Pictoral representation of the overall architecture

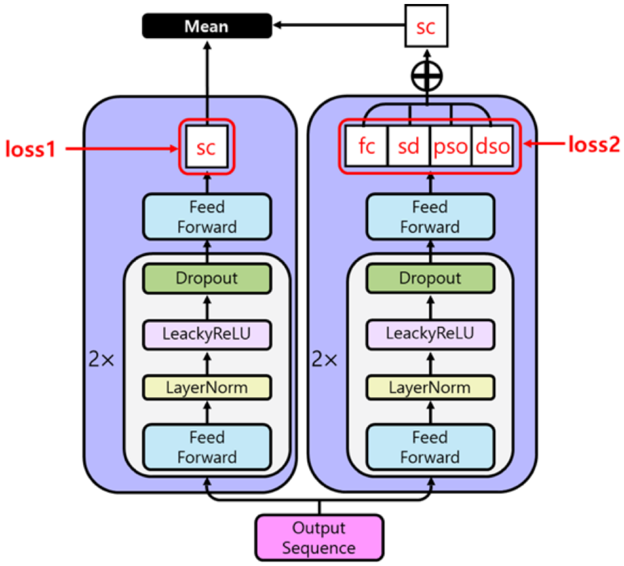

Figure S10: Pictoral representation of the overall model

model is constructed as the log of the sum of MAEs of outputs of two Readout layers.

S9.4 Features

An atomic pair feature is composed of two atoms' properties and pair properties. (See Figure S11) For atomic properties, the position of atom, atomic number and atomic charge, available in QM9 dataset are used and distance between them and type of coupling are for the pair properties. Those values are embedded and concatenated to build feature vector. Unlike the sum operation, concatenation is not a commutative operation so our feature vectors are dependent on the order of atoms in a pair. To make the feature vector independent of the order of atoms, test time augmentation or data augmentation can be applied, but in the data set, the order of atoms in a pair are sorted. Therefore, we use none of them. For clarity, it should be noticed that this order-dependency does not necessarily lead to permutation dependency of atomic pairs belonging to molecules.

S9.4.1 Data Augmentation

As described in the previous section, positions of atoms are used in input sequences, therefore rotational and translational invariances are not conserved in our model. Although both invariances are not mathematically preserved, by training augmented data, we make parameters of our model guided to have pseudo-invariance in a certain range of translational and rotational changes.

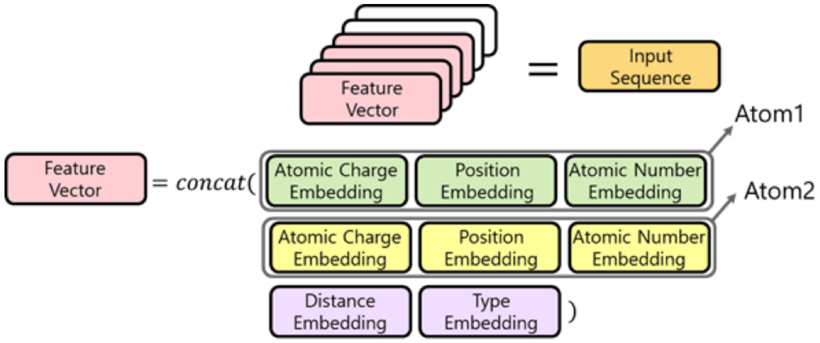

Figure S11: Pictoral representation of the input features

Fortunately, positions of atoms belonging to the QM9 set do not have extreme values, which means this kind of less rigorous strategy can cover all the test cases in the competition. If we need to cover general molecular geometry, this kind of strategy might not work. Generation of noise can be done in every epoch with low computational cost, the size of the original training database being enlarged 10 times with randomly selected noise. The selected noise is not changed during the training. The translational perturbations on each axis and angle of rotational perturbations are sampled from a Normal distribution. By this augmentation of the training data, we observed that the trained model recorded small enough disagreements with both changes and a better score in leaderboard. Mean and standard deviation values for both translational and rotational perturbation can be found in Table S8.

S9.4.2 Dummy types

Some of the geometric information of molecule is missing in the input sequences because not all atomic pairs are included. It may cause an incomplete description of chemical circumstances. In order to overcome this limit, dummy pairs are introduced. The dummy pairs are a part of the input sequences but do not have coupling constant values. The predicted values of dummy types are not used to evaluate the loss but the feature vectors of dummy types participate in process of building latent space even for meaningful pairs. By this, the geometric information of non-activated pairs can be included in the input sequence. Thousands of pair types exists in the given training set. Among them, 7 additional atomic types are included in input sequences: 1JHO, 1JCO, 1JCN, 1JNO, 1JCC, 1JNN, and 1JFC. The selection of these dummy pairs is a tunable hyperparameter but due to a lack of computing power, we did not test various combinations of dummy types. We choose to add neighboring types first because nearby pairs may have a strong impact on chemical circumstances. If more dummy types are added, more geometric information can be included in input sequences but an increase of computational costs follows as the length of sequence increases.

S9.5 Training and Ensembling

In order to achieve a high score, we employed an ensemble technique and pseudo-labeling. An ensemble technique is to merge the results of various models and pseudo-labeling is to use new dataset which contains unlabeled data whose label is assumed as the results of previously trained model. We originally planned to use a seed ensemble which uses a number of identical models with different random seed numbers but because of inequalities of teammates' computing resources. Therefore, models with different N values (in Figure S9) and epochs are used. An overall training process is illustrated in Figure S12. At first stage, 15 models are trained using only training dataset with dropout. At second stage, parameters of models were fine-tuned by turning off the dropouts. By using models trained up to the second phase, labels for test sets (so-called pseudo-label) are predicted. To minimize error of pseudo-label, results of 4-8 trained models up to the second phase are employed. In the third phase, newly constructed training sets which including both original training dataset and pseudo-labeled test set are used. Because of due date of competition, we only take 8 models and further trained with the expanded datasets. At phase 3 and 4, 20 epochs are progressed. To mitigate the unpredicted bias from pseudo-labeling, at the last phase, the models are trained with only the original training dataset. Adam optimizer, typical choice, is employed with gradient clipping. Linear warmup and linear decay are used for the stable convergence of training. All weights are initialized with uniform distribution (mean=0.0, std.=0.02) and 0 is used for initial bias values.

S9.5.1 Specification of models

Training in each ensemble is composed of 4 different steps. First step, training is performed with training set data and dropout. In the second step, further training is proceeded without dropout. Using the obtained model from the second step, the coupling constants for testing set are predicted; these predicted values are called pseudo label. The pseudo-labeled data in addition to training data is used in the third training step. To mitigate overfitting, further training is performed with only training set.

Output values of each type are normalized because each type shows different distribution of target values.

S9.6 Performance

Our final model has around 75M parameters. With two V100 graphic cards, training the model takes around 2 days.

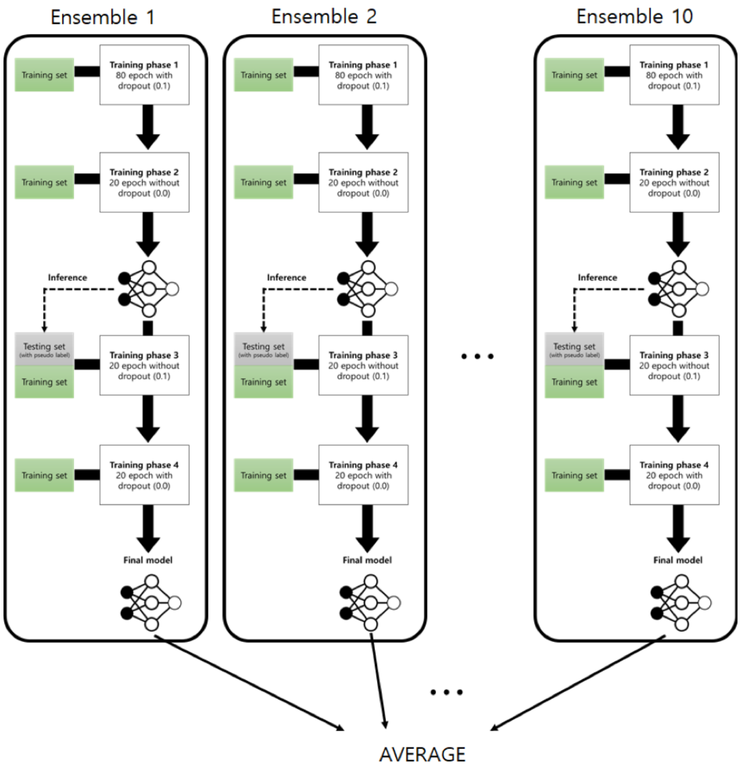

Figure S12: Pictoral representation of the training procedure

S10 Solution 4

S10.1 Model Architecture

The model we used is a development of the Graph Attention Network [28] architecture with Gated Residual Connections [29]. The model operates on molecular graphs (nodes being the atoms, and edges being the bonds), augmented by artificial links between the nodes representing atoms 2 and 3 bonds apart in the molecular structure. Both graph nodes and graph edges hold feature vectors (in contrast to the more standard approach where only the nodes hold feature vectors). Size of the node and edge feature vectors are equal in each layer. The network is implemented in the PyTorch [30] framework, using the Deep Graph Library [31], which requires all graph edges to be directed. We therefore represent each bond (as well as each 1-3 and 1-4 link) as a pair of graph edges (one in each direction).

The network consists of multiple layers, where each layer updates both the node and the edge feature vectors through the following steps:

1. Fully connected layers applied to nodes and edges:

$$\eta^l = W^l_{node} n^{l-1}, \epsilon^l = W^l_{edge} e^{l-1}$$

   where n^{l-1} and e^{l-1} represent the values of the node and edge embeddings from the previous layer, while η and ϵ represent intermediate values used within the rest of the layer.

2. Attention based convolutional update applied to each node:

$$\alpha^l_{ij} = softmax_j(\sigma(A^l[\eta^l_i \parallel \epsilon^l_{ij} \parallel \eta^l_j]))$$

$$n^l_i = \sigma(\sum_j (\alpha^l_{ij} \eta^l_i \epsilon^l_{ij}))$$

   where σ is a non-linearity (in the final model we used the Leaky Rectified Linear Unit with the slope set to 0.2). Furthermore, we employ the multi-head extension to the attention mechanism (as described in [28]), where multiple independent attention mechanisms are applied, and their results concatenated:

$$n^l_i = \Big\Vert_{k=1}^{K} \sigma(\sum_j (\alpha^l_{ijk} \eta^l_{ik}) \epsilon^l_{ijk})$$

3. Fully connected update to each edge, based on the concatenation of the source node feature vector, edge feature vector, and the destination node feature vector:

$$e^l_{ij} = W^l_{triplet}[n^l_i \parallel \epsilon^l_{ij} \parallel n^l_j]))$$

4. At the end of each layer we apply layer normalization [32] to node and edge feature vectors.

Each layer is followed by a Gated (Parametric) Residual connection [29], on both the node and the edge feature vectors. The outputs of the residual values are passed through Parametric Rectified Linear Units [33].

The final model is made up of an embedding layer, eight residual layers, and the two fully connected output layers applied to edge feature vectors of the final layer. Embedding size for both the nodes and the edges in each residual layer is 1152, and the number of heads is 24 (each head accounting for 48 parameters). The output sizes of the two final fully connected output layers were 512 and 8 (one output for each of the coupling types).

Coupling constant between two atoms are predicted by taking the corresponding output from the edges linking the graph nodes representing the two atoms. For each pair of atoms the graph will incorporate two such edges (one in each direction, due to the directed nature of the graph). During training, we treat these as two independent predictions (calculating and back-propagating the loss as if these were two data points). In prediction mode we output the average of these two predictions.

S10.2 Features

In addition to the molecular graph structure (atoms and bonds as inferred by OpenBabel [34]) the model uses a number of atom and bond features. For atoms we use: electronegativity, first ionization energy, and electron affinity for each atom type, as well as atom Mulliken charge taken from the QM9 dataset [35, 36]. For edges we use features: bond length, bond angle (for artificial 1-3 edges), and the dihedral angle (for artificial 1-4 edges). All features were standardized based on the training set statistics. Labels were standardised for all models, except those used for predicting 1JHC, where we only subtract the mean (which we empirically determined to produce better results).

S10.3 Training and ensembling

We trained the model using a modified version of the LAMB optimizer [37], where we decouple the weight decay term from the trust region calculations, similarly to the AdamW modification of the Adam optimizer [38]. The training was done using a mini-batch size of 80 molecules. Models were first pre-trained to jointly predict the scalar coupling constants for all coupling types. In the fine-tuning step we train separate models for JHC and JHH types, and continue training the joint model for the JHN types. The training was done using the Mean Absolute Error loss.

During training we used the following dynamic learning rate schedule:

- 30 epoch cycle, linearly varying the learning rate from 0.001 to 0.01 and back (pre-train phase)
- 70 epochs with a constant learning rate of 0.001 (pre-train phase)
- Constant learning rate of 0.001 until convergence; 90-100 cycles, depending on coupling type (fine tuning phase)

The model was regularized using weight decay set to 0.05 for the first 30 epochs and 0.01 afterwards. Final model parameters are derived by running Stochastic Weight Averaging [39] over the models from the final 25 epochs of training. Final predictions are produced as the mean of the outputs from two folds (two sets of models, both using the same architecture, but with a different train/validation split, with 90% of the data used in training, and 10% used for validation). Each of the two model sets consists of 6 models:

- a model per coupling type, for each of 1JHC, 2JHC, 3JHC, 2JHH, 3JHH coupling types
- 1 model specialized for 1JHN, 2JHN, and 3JHN coupling types

S10.4 Performance

The training procedure for the final ensemble took 200 GPU hours on systems with 2080Ti cards. The ensemble achieved a score of -3.18667 on the public test set, and -3.18085 on the private test set provided by the competition. In order to illuminate the architecture's performance, in addition to the final model (denoted FULL) we also benchmarked a single model trained to predict all of the coupling type interaction jointly (denoted SINGLE), and an ensemble where single model is specialized for each coupling type (denoted TYPE). The results are presented in Table S5.

Table S5: Scores achieved by different ensembling choices.

Model	Private score	Public score	Train time (GPU h)
FULL	-3.18085	-3.18667	200
PER-TYPE	-3.13853	-3.14362	85
SINGLE	-2.96183	-2.96443	24

S11 Solution 5

Our model is an ensemble model built from 16 graph-based deep learning models. Our base models are inspired from MatErials Graph Network (MEGNet)[40] which is an architecture used to predict properties of either molecules or crystals. In the following we will describe our best single model.

S11.1 Input features

We created features from the raw atom elements and coordinates using OpenBabel[34]. OpenBabel provides numerous chemical features for each atoms, bonds, and for the whole molecule, which are all included in our input features. We also added translation and rotation invariant features such as ACSFs[41], distances of bonds, angle between bonds and the raw coordinates. Random rotations on

the molecule coordinates were applied as a way of data augmentation. The most important features sorted by a permutation feature importance are the following:

- **Atom**: size of smallest ring that contains the atom, heteroatom valence, number of ring connection to the atom, average angle of bonds connected to the atom, whether a neighboring atom has an unsaturated bond to a third atom, count of hydrogen bound to the atom.
- **Bond**: angle formed by the neighboring bond with the closest atom, minimum distance of neighboring bond, bond distance, scalar coupling type.
- **Molecule**: number of atoms, number of non-hydrogen atoms.

We also noticed that the most important features of our preliminary models, according to a permutation feature importance, were the ring topology in the molecule and the angles between two bonds. This inspired us to modify MEGNet by adding two operations related to rings and bond-bond angles. We will describe these modifications below.

S11.2 Model architecture

Our models consists of three stages :

1. a preprocessing operation that transforms molecular, bonds and atomic features into a graph representation.
2. multiple steps of a graph update operation.
3. a readout operation that transform the graph representation to a set of scalar coupling values.

The architecture is described in Figure S13.

S11.2.1 Preprocessing operation

The preprocessing operation builds a graph representation of the molecule. Each atom in the molecule represents a node in the graph. We choose to represent the molecule with a dense graph rather that limiting the edges to chemical bonds. This choice helped the information flows better in our network. Each node, edge and the whole graph are associated to a state vector. To build each state vector, we apply a multi-layer perceptron to the features of each considered object.

S11.2.2 Graph update operation

The graph update operation takes a graph as input and outputs the same graph structure with updated state vector values. It consists of three steps :

1. update the edge state vectors

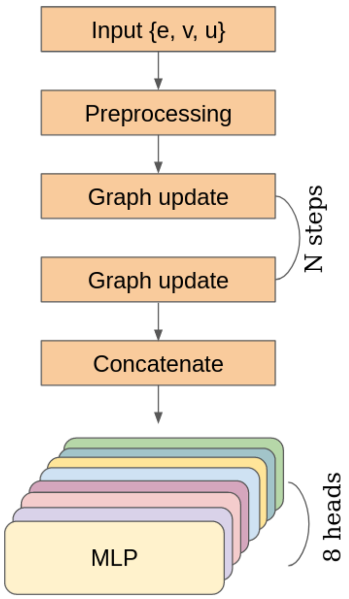

Figure S13: Best single model architecture

2. update the node state vectors

3. update the global state vector

We denote $V = \{\mathbf{v}_i\}_{i=1:N_v}$ the set of node vectors, with N_V the node count, $E = \{\mathbf{e}_k\}_{k=1:N_E}$ the set of edge vectors, with N_E the edge count, and $\mathbf{u}$ the global graph vector. $G = (V, E, \mathbf{u})$ is the input graph representation and $G' = (V', E', \mathbf{u}')$ is the output graph representation of the graph update operation.

In the following, we denote all ϕ functions as multi-layer perceptron with two hidden layers, SoftPlus activation and LayerNormalization that takes a vector as input and outputs another vector. If two ϕ functions share the same subscript it means that the multi-layer perceptrons share their parameters. If the ϕ function has no subscript it means that its parameters are not shared with any other multi-layer perceptron.

All ψ functions are vector aggregation functions that outputs one vector from a set of vectors. In our architecture, all ψ aggregations are attention functions that takes two parameters :

- a set of vectors to aggregate, which is used as the keys and values in the attention mechanism
- a vector which is used as query in the attention mechanism

The $\bigoplus$ operator is the vector concatenation operator.

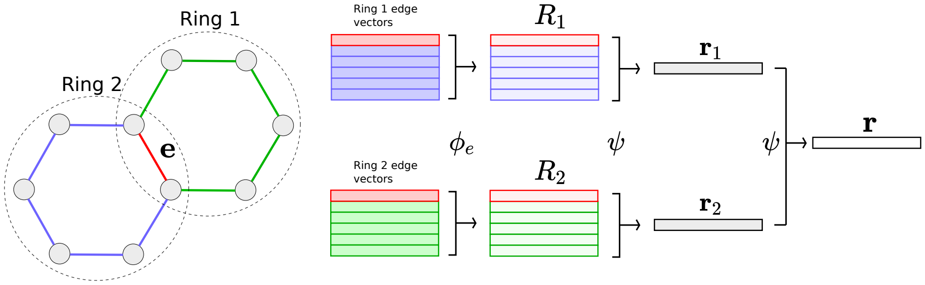

Figure S14: Computation of $\mathbf{r}$ with two rings

Edge vector update The edge vector update consists of four operations. In the following we denote $\mathbf{e}$ the edge vector to be updated and b the bond associated with it.

Firstly we introduce an intermediary vector $\mathbf{r}$ which is an aggregation of the edge vectors contained in all rings b is a part of, where a ring denote a simple cycle of atoms and chemical bonds in a molecule. The purpose of $\mathbf{r}$ is to allow a better flow of the rings related information. We proposed this intermediate vector because we observed that rings features had an high impact on preliminary scalar coupling models.

We denote N_R the number of rings containing b in the molecule. For the ith ring containing b, $R_i = \{\phi_e(\mathbf{e}_k)\}_{k=1:N_{R_i}}$ is the processed edge vectors of the ring. The vector $\mathbf{r}$ is computed as follows :

$$\mathbf{r}_i = \psi\Big(R_i, \phi_e(\mathbf{e})\Big)$$
$$\mathbf{r} = \psi\Big(\{\mathbf{r}_i\}_{i=1:N_R}, \phi_e(\mathbf{e})\Big)$$

$\mathbf{r}_i$ is an aggregated edge vector along the ith ring containing b and $\mathbf{r}$ is an aggregated edge vector along in all the rings containing b. An example of such computation is displayed in Figure S14.

Secondly we introduce another intermediary vector $\mathbf{a}$ which is an aggregation of neighboring edge vectors and local geometric features. The purpose of $\mathbf{a}$ is firstly to allow to integrate angular edge-edge features into our architecture and secondly to provide a better flow of the edge-edge information. We proposed this intermediate vector because we observed that the engineered edge feature "angles between one edge and the edges formed with the closest atom" feature had an high impact on our preliminary scalar coupling models.

We denote $A = \{(\mathbf{e}_i^1, \mathbf{e}_i^2)\}_{i=1:N_V-2}$ is all couples of edges formed between an atom in the molecule and the two atoms in $\mathbf{e}$. $\mathbf{f}_i$ is the feature vector characterising the triangle of edges $(\mathbf{e}, \mathbf{e}_i^1, \mathbf{e}_i^2)$ which is 5-dimensional and contains the 3 angles in the triangle as well as the length of edges $\mathbf{e}_i^1$ and $\mathbf{e}_i^2$. The vector $\mathbf{a}$ is defined as follows :

$$\mathbf{a} = \psi\Big(\Big\{\phi(\mathbf{f}_i) \bigoplus \phi_e(\mathbf{e}) \bigoplus \phi_e(\mathbf{e}_i^1) \bigoplus \phi_e(\mathbf{e}_i^2)\Big\}_{i=1:N_V-2}, \phi_e(\mathbf{e})\Big)$$

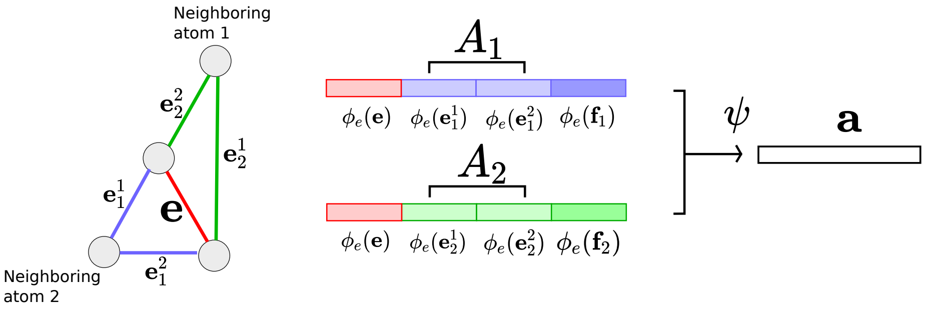

Figure S15: Computation of $\mathbf{a}$ for the edge $\mathbf{e}$ in a four node system.

An example of such computation is displayed in Figure S15.

Finally we compute the updated edge vector $\mathbf{e}'$. We denote the $\mathbf{v}_1$ and $\mathbf{v}_2$ the first and second atom vector of $\mathbf{e}$.

$$\mathbf{e}' = \phi\Big(\mathbf{r} \bigoplus \mathbf{a} \bigoplus \phi_e(\mathbf{e}) \bigoplus \phi_u(\mathbf{u})\Big) + \phi_{atom}(\mathbf{v}_1) + \phi_{atom}(\mathbf{v}_2) + \mathbf{e} \tag{S16}$$

$\mathbf{e}'$ contains a skip connection to enable deeper and faster model training.

Node update We denote $\mathbf{v}$ the node vector to be updated, $\mathbf{v}_i$ the ith node vector different from $\mathbf{v}$ and $\mathbf{e'}_i$ the updated edge vector between the nodes associated with $\mathbf{v}$ and $\mathbf{v}_i$. Let $C = \{\mathbf{v}_i \bigoplus \mathbf{e'}_i\}_{i=1:N_V-1}$ be the set of concatenated node and updated edge vectors. The vector $\mathbf{v}'$ is defined as follows :

$$\mathbf{v}_{agg} = \psi\Big(C, \phi_v(\mathbf{v})\Big)$$
$$\mathbf{v}' = \phi_s\Big(\mathbf{v}_{agg} \bigoplus \phi_v(\mathbf{v}) \bigoplus \phi_u(\mathbf{u})\Big) + \mathbf{v}$$

As for the edge update, we integrate a skip connection.

Global state update We denote $V' = \{\mathbf{v'}_k\}_{k=1:N_V}$ the set of updated node vectors and $E' = \{\mathbf{e'}_k\}_{k=1:N_E}$ the set of updated edges vectors. The updated global state vector is defined as follows :

$$\mathbf{u}_{agg\_edge} = \psi\Big(E', \phi_u(\mathbf{u})\Big)$$
$$\mathbf{u}_{agg\_node} = \psi\Big(V', \phi_u(\mathbf{u})\Big)$$
$$\mathbf{u}' = \phi\Big(\mathbf{u}_{agg\_edge} \bigoplus \mathbf{u}_{agg\_node} \bigoplus \phi_u(\mathbf{u})\Big) + \mathbf{u}$$

As for the edge and node update, we integrate a skip connection.

S11.2.3 Readout stage

To predict a scalar coupling associated with an edge, we concatenated the edge vector, the two node vectors associated with the edge, and the global state vector and passed it through multi-layer perceptron to generate the final output. Since there was 8 different types of scalar coupling, our model had 8 different multi-layer perceptrons to enforce a different readout for each type.

S11.3 Training and ensembling

We trained our model with the Adam[20] optimizer with a fixed learning rate and the original article default parameters, for about 150 epochs then reduced the learning rate by a factor 2 each 3 epochs for 15 epochs.

The dimension of our graph vectors was set at 300. Our batch size was 20, a relatively small number due to the high memory requirements of the computation of the neighboring edge vectors aggregation.

S11.3.1 Experiments and model variations

We observed that even models with a modest score could help to contribute to a better overall performance with an ensemble model. As such we tried various architectures with different modifications. The following experiments were tested and integrated as base models:

- Different activation functions in our multi-layer perceptron: Softplus provided a boost of performance in comparison to a ReLU baseline.
- Normalization: LayerNormalization worked better that BatchNormalization in our experiments and helped improve convergence.
- In the readout stage, rather that using only the edge and two nodes associated with a scalar coupling, we concatenated all the edges and nodes vectors in the chemical bond path of the two atoms we compute the scalar coupling. We observed faster training with this method but could not integrate it in our best single model in time.
- We iterated with different number of graph update operations or different number of hidden layers.

S11.3.2 Ensemble learning

Ensemble learning is a technique that aggregates multiple models into a single one to obtain a better performance. It has become a standard in machine learning competitions. To build our final prediction, we fitted a linear model and a gradient boosting model that we averaged. Rather than using only our 16 base models in this ensemble, we also integrating intermediary models checkpoints of those 16 models to further improve the performance. In the end there was about 50 input models in the ensemble.

S11.4 Improvements

We can think of few improvements for our best single model architecture:

- Prune the amount of edges considered in the neighboring edge vectors aggregation by keeping only the edges closest to the updated edge. This would greatly reduce the memory consumption of the architecture, allow a bigger batch size and fasten the model. We suppose that this would not degrade the performance as the angular features that had high permutation importance in our preliminary models were related to the closest edges.

- Integrate the full chemical bond path in the readout stage mentioned in S11.3.1 to increase the training speed as it proved efficient on other similar architectures.

S12 Solution 12

S12.1 Introduction

There has been extensive work recently using graph neural networks for predicting properties of molecular systems. Many problems along this direction require the use of configuration information (i.e., the positions of all atoms in a molecule.) A key challenge in applying machine learning techniques to these problems is that of capturing local geometric information (such as bond or torsion angles), while preserving symmetries of the overall system. Symmetries such as rotation and translation invariance are fundamental properties of molecular systems, and therefore must be exactly preserved in order for a machine learning architecture to effectively use training data and make meaningful predictions.

Our recent paper proposed the COvaRiant MOleculaR Artificial Neural neTwork *(Cormorant)* to help solve these issues [42]. *Cormorant* uses spherical tensors as activation to encode local geometric information around each atom's environment. A spherical tensor is an object F^ℓ which transforms in a predictable way under a rotation. Specifically, for a rotation $R \in \mathrm{SO}(3)$, there is a matrix $D^\ell(R)$, such that $F^\ell \to D^\ell(R) F^\ell$. Here, $D^\ell(R)$ is known as a Wigner-D matrix, and ℓ is known as the order of the representation.

A tensor with $\ell = 0$ is a scalar quantity (that is, invariant under rotations), whereas $\ell = 1$ is a vector quantity such as a dipole, and $\ell = 2$ is a second-order tensor like a quadrupole, and so on. This structure is well known in physics. For example, the multipole expansion is a decomposition of an electrostatic potential $V(\mathbf{r}) = \sum_{\ell=0}^{\infty} Q^\ell Y^\ell(\hat{\mathbf{r}}) / r^{\ell+1}$ into multipole moments Q^ℓ and spherical harmonics $Y^\ell(\hat{\mathbf{r}})$ oriented in the direction of the vector $\mathbf{r}$. For a more in-depth discussion of spherical tensors, we point the reader to Ref. [42].

The use of spherical tensors allows for a network architecture that is "covariant to rotations." This means that if level s, a rotation is applied to all activations $F^{s,\ell} \to D^\ell(R) F^{s,\ell}$, then at the next level $s+1$, all activations will

automatically inherit that rotation so $F^{s+1,\ell} \to D^{\ell}(R) F^{s+1,\ell}$. As such, a rotation to the input of *Cormorant* will propagate through the network to ensure the output transforms as well. In this way, we can capture local geometric information, but can still maintain the desired transformation properties under rotations.

A key point is that the quantum algebra (SU(2)) used in the definition of NMR couplings is for our purposes equivalent (homomorphic) to the SO(3) algebra used in *Cormorant*[1]. The J-Coupling Hamiltonian

$$H = 2\pi \mathbf{I}_i \cdot \mathbf{J}_{ij} \cdot \mathbf{I}_j$$

describes the couplings $\mathbf{J}_{ij}$ between spin operators $\mathbf{I}_i$. The spin operators are themselves objects that generate the Lie algebra SU (2), and thus transform according to Wigner-D matrices. *Cormorant* therefore naturally incorporates the structure of J-Coupling Hamiltonian, and we expect is a natural platform learning NMR couplings.

S12.2 Model architecture

We now briefly summarize our *Cormorant* architecture; for a more in-depth description of our architecture, see the original paper [42].

The structure of of *Cormorant* is similar to a graph neural network, with the key difference that vertex activations F_i and edge activations G_{ij} for atoms i, j are promoted to lists of spherical tensors $F_i = \left(F_i^0, \ldots, F_i^{\ell_{\max}}\right)$ and $G_{ij} = \left(G_{ij}^0, \ldots, G_{ij}^{\ell_{\max}}\right)$, where $F_i^\ell \in \mathbb{C}^{(2\ell+1)\times n_\ell}$ and $G_{ij}^\ell \in \mathbb{C}^{(2\ell+1)\times n_\ell}$ are spherical tensors of order ℓ, and n_ℓ is the multiplicity (number of channels) of the tensor.

In order to maintain covariance, we must carefully choose our non-linearity. The core non-linearity in *Cormorant* is dictated by the structure of the $D^\ell(R)$ matrices, and is known as the Clebsch-Gordan product [CG]. We express the CG product of $\otimes_{\text{cg}}$ of two spherical tensors as:

$$[A_{\ell_1} \otimes_{\text{cg}} B_{\ell_2}]_\ell = \bigoplus_{\ell=|\ell_1-\ell_2|}^{\ell_1+\ell_2} C_{\ell_1\ell_2\ell}\left(A_{\ell_1} \otimes B_{\ell_2}\right)$$

where $\otimes$ denotes a Kronecker product, and $C_{\ell_1\ell_2\ell}$ are the famous Clebsch-Gordan coefficients [CG].

Our vertex activations F_i^s at level s are chosen to be

$$F_i^s = \left[F_i^{s-1} \oplus \left(F_i^{s-1} \otimes_{\text{cg}} F_i^{s-1}\right) \oplus \left(\sum_j G_{ij}^s \otimes_{\text{cg}} F_j^{s-1}\right)\right] \cdot W_{s,\ell}^{\text{vertex}}$$

where $\oplus$ denotes concatenation and $W_{s,\ell}^{\text{vertex}}$ is a linear mixing layer that acts on the multiplicity index. We choose the edge activations to have the form of

[1]More precisely, SU(2) is a double cover of SO(3), and both groups share a Lie algebra.

$G_{ij}^{s,\ell} = g_{ij}^{s,\ell} \times Y^{\ell}(\hat{\mathbf{r}}_{ij})$, where $\mathbf{r}_{ij}$ is a vector pointing from atom i to atom j. The scalar-valued edge terms are given by

$$g_{ij}^{s,\ell} = \mu^{s}(r_{ij}) \left[\left(g_{ij}^{s-1,\ell} \oplus \left(F_i^{s-1} \cdot F_j^{s-1} \right) \oplus \eta^{s,\ell}(r_{ij}) \right) \cdot W_{s,\ell}^{\text{edge}} \right]$$

with $\mu^{s}(r_{ij})$ a learnable mask function, $\eta^{s,\ell}(r_{ij})$ a learnable set of radial basis functions, and $W_{s,\ell}^{\text{edge}}$ a linear layer along the multiplicity index.

We iterate this architecture for $s = 0, \ldots, s_{\text{max}}$. Finally, at the last layer of *Cormorant*, we take the $\ell = 0$ component of the edge $g_{ij}^{s_{\text{max}}\ell}$, and use it as a prediction of the J-coupling.

S12.3 Training and ensembling

To predict J-coupling constants, we first observed that each J-coupling was a rotationally invariant feature on pairs of atoms. Consequently, it was natural to read the values of the J-couplings off the values of $g_{ij}^{s,\ell}$. We therefore constructed a neural net with five cormorant layers, with a maximum ℓ value of 3. In each layer, we used 48 vertex activations for each value of ℓ. We then took a linear combination of the values of $g_{ij}^{s,\ell}$ for each layer and took a learned linear combination of the values as our prediction for the scalar coupling constant. Initial features were constructed using the radial distance between atoms, the atomic identity, and the atomic charge. In general, almost exactly the same network was used as for the QM9 tests in the Cormorant paper [42]. We also used the Mulliken charges as initial features for pretraining. Our training proceeded in three stages.

1. Three nets were trained on the 1J, 2J, and 3J couplings with the Mulliken Charges on an internal split on the training data.

2. Eight nets were trained on the 1JHC, 1JHN, 2JHH, 2JHC, etc. couplings with the Mulliken Charges on an internal split on the training data, with weights initialized to be the values in stage 1.

3. Each of the nets in stage 2 was trained on the full dataset, without access to the Mulliken charges.

The internal split was constructed using an 80/10/10 train/validation/test split on the training dataset provided in the Kaggle competition. The model was optimized using the AMSGrad optimizer for 200 epochs. The learning rate was varied using cosine annealing. For stage one the initial and final learning was $5 * 10^{-4}$ and $5 * 10^{-6}$, and for stages two and three the initial and final learning rates were $3 * 10^{-4}$ and $3 * 10^{-6}$, respectively. We repeated stages two and three with different random seeds in attempt to average over multiple trained networks, however this had negligible effect on the results.

In table S6, we give the final results on the Kaggle Test/Train split. We achieve considerably stronger results for the 2J and 3J couplings than for the 1J couplings. However, we were pleasantly surprised by the minimal fine-tuning

Table S6: Performance

Dataset	1JHC	1JHN	2JHH	2JHC	2JHN	3JHH	3JHC	3JHN
Training	-2.736	-4.834	-4.366	-3.481	-5.4889	-4.632	-3.275	-5.557
Test	-1.768	-2.224	-3.584	-2.856	-3.312	-3.552	-2.688	-3.496

required to get results close to state of the art. Future directions include directly connecting the tensors learned by cormorant with the tensor-valued data in NMR experiments to provide more detailed inferences.

S13 Code and data

Code and data is available on `http://osf.io/kcaht` and `http://github.com/larsbratholm/champs_kaggle`. These contain the following

- List of molecules removed as they did not contain hydrogens.
- List of molecules removed due to one or more couplings being outliers.
- List of training and test molecules.
- Script to convert QM9 extended XYZ-files into both regularly formatted XYZ-files as well as input files for Gaussian NMR computations.
- Script to create the Kaggle dataset from the output files of Gaussian NMR computations.
- Archive of all extended XYZ-files of QM9 that passed the consistency check of the original paper.
- Archive of all regular formatted XYZ files parsed from the extended XYZ file format of the QM9 dataset.
- Archive of all Gaussian input files.
- Archive of all Gaussian output files.
- Archive of the Kaggle dataset.
- Archive of the top 400 submissions.
- Script to create an ensemble from the top submissions.
- Script to create the plots used in the paper and SI.
- Code used by the 1st, 2nd, 3rd, 4th, 5th and 12th placed teams to create their models.

References

[1] Raghunathan Ramakrishnan, Pavlo O Dral, Matthias Rupp, and O Anatole von Lilienfeld. Quantum chemistry structures and properties of 134 kilo molecules. *Scientific Data*, 1, 2014.

[2] P. J. Stephens, F. J. Devlin, C. F. Chabalowski, and M. J. Frisch. Ab initio calculation of vibrational absorption and circular dichroism spectra using density functional force fields. *The Journal of Physical Chemistry*, 98(45):11623–11627, 1994.

[3] R. Ditchfield, W. J. Hehre, and J. A. Pople. Self-consistent molecular-orbital methods. ix. an extended gaussian-type basis for molecular-orbital studies of organic molecules. *The Journal of Chemical Physics*, 54(2):724–728, 1971.

[4] Michael J. Frisch, John A. Pople, and J. Stephen Binkley. Self-consistent molecular orbital methods 25. supplementary functions for gaussian basis sets. *The Journal of Chemical Physics*, 80(7):3265–3269, 1984.

[5] W. J. Hehre, R. Ditchfield, and J. A. Pople. Self—consistent molecular orbital methods. xii. further extensions of gaussian—type basis sets for use in molecular orbital studies of organic molecules. *The Journal of Chemical Physics*, 56(5):2257–2261, 1972.

[6] R. Krishnan, J. S. Binkley, R. Seeger, and J. A. Pople. Self-consistent molecular orbital methods. xx. a basis set for correlated wave functions. *The Journal of Chemical Physics*, 72(1):650–654, 1980.

[7] F. Pedregosa, G. Varoquaux, A. Gramfort, V. Michel, B. Thirion, O. Grisel, M. Blondel, P. Prettenhofer, R. Weiss, V. Dubourg, J. Vanderplas, A. Passos, D. Cournapeau, M. Brucher, M. Perrot, and E. Duchesnay. Scikit-learn: Machine learning in Python. *Journal of Machine Learning Research*, 12:2825–2830, 2011.

[8] Martín Abadi, Ashish Agarwal, Paul Barham, Eugene Brevdo, Zhifeng Chen, Craig Citro, Greg S. Corrado, Andy Davis, Jeffrey Dean, Matthieu Devin, Sanjay Ghemawat, Ian Goodfellow, Andrew Harp, Geoffrey Irving, Michael Isard, Yangqing Jia, Rafal Jozefowicz, Lukasz Kaiser, Manjunath Kudlur, Josh Levenberg, Dandelion Mané, Rajat Monga, Sherry Moore, Derek Murray, Chris Olah, Mike Schuster, Jonathon Shlens, Benoit Steiner, Ilya Sutskever, Kunal Talwar, Paul Tucker, Vincent Vanhoucke, Vijay Vasudevan, Fernanda Viégas, Oriol Vinyals, Pete Warden, Martin Wattenberg, Martin Wicke, Yuan Yu, and Xiaoqiang Zheng. TensorFlow: Large-scale machine learning on heterogeneous systems, 2015. Software available from tensorflow.org.

[9] Pauli Virtanen, Ralf Gommers, Travis E. Oliphant, Matt Haberland, Tyler Reddy, David Cournapeau, Evgeni Burovski, Pearu Peterson, Warren Weckesser, Jonathan Bright, Stéfan J. van der Walt, Matthew Brett, Joshua Wilson, K. Jarrod Millman, Nikolay Mayorov, Andrew R. J. Nelson, Eric Jones, Robert Kern, Eric Larson, CJ Carey, İlhan Polat, Yu Feng, Eric W. Moore, Jake Vand erPlas, Denis Laxalde, Josef Perktold, Robert Cimrman, Ian Henriksen, E. A. Quintero, Charles R Harris, Anne M. Archibald, Antônio H. Ribeiro, Fabian Pedregosa, Paul van Mulbregt, and SciPy 1. 0 Contributors. SciPy 1.0: Fundamental Algorithms for Scientific Computing in Python. *Nature Methods*, 2020.

[10] I Borg and P Groenen. P.(1997) modern multidimensional scaling. theory and applications.

[11] Joseph B Kruskal. Nonmetric multidimensional scaling: a numerical method. *Psychometrika*, 29(2):115–129, 1964.

[12] Joseph B Kruskal. Multidimensional scaling by optimizing goodness of fit to a nonmetric hypothesis. *Psychometrika*, 29(1):1–27, 1964.

[13] Will Gerrard, Lars A Bratholm, Martin J Packer, Adrian J Mulholland, David R Glowacki, and Craig P Butts. Impression–prediction of nmr parameters for 3-dimensional chemical structures using machine learning with near quantum chemical accuracy. *Chemical Science*, 11(2):508–515, 2020.

[14] Greg Landrum. Rdkit: Open-source cheminformatics. `http://www.rdkit.org`.

[15] Justin Gilmer, Samuel S. Schoenholz, Patrick F. Riley, Oriol Vinyals, and George E. Dahl. Neural message passing for quantum chemistry. In *Proceedings of the 34th International Conference on Machine Learning - Volume 70*, ICML'17, page 1263–1272. JMLR.org, 2017.

[16] Mohammed Alsuhaibani, Takanori Maehara, and Danushka Bollegala. Joint learning of hierarchical word embeddings from a corpus and a taxonomy. In *Automated Knowledge Base Construction (AKBC)*, 2019.

[17] Ashish Vaswani, Noam Shazeer, Niki Parmar, Jakob Uszkoreit, Llion Jones, Aidan N Gomez, Łukasz Kaiser, and Illia Polosukhin. Attention is all you need. In *Advances in neural information processing systems*, pages 5998–6008, 2017.

[18] Jimmy Lei Ba, Jamie Ryan Kiros, and Geoffrey E. Hinton. Layer normalization, 2016.

[19] Adam Paszke, Sam Gross, Francisco Massa, Adam Lerer, James Bradbury, Gregory Chanan, Trevor Killeen, Zeming Lin, Natalia Gimelshein, Luca Antiga, et al. Pytorch: An imperative style, high-performance deep learning library. In *Advances in Neural Information Processing Systems*, pages 8024–8035, 2019.

[20] Diederik P. Kingma and Jimmy Ba. Adam: A method for stochastic optimization, 2014.

[21] Ilya Loshchilov and Frank Hutter. Sgdr: Stochastic gradient descent with warm restarts, 2016.

[22] Ashish Vaswani, Noam Shazeer, Niki Parmar, Jakob Uszkoreit, Llion Jones, Aidan N Gomez, Łukasz Kaiser, and Illia Polosukhin. Attention is all you need. In *Advances in neural information processing systems*, pages 5998–6008, 2017.

[23] Jeremy Howard et al. fastai. `https://github.com/fastai/fastai`, 2018.

[24] Martin A. Fischler and Robert C. Bolles. Random sample consensus: A paradigm for model fitting with applications to image analysis and automated cartography. *Commun. ACM*, 24(6):381–395, June 1981.

[25] Xin Dang, Hanxiang Peng, Xueqin Wang, and Heping Zhang. Theil-sen estimators in a multiple linear regression model. *Olemiss Edu*, 2008.

[26] Kristof T Schütt, Farhad Arbabzadah, Stefan Chmiela, Klaus R Müller, and Alexandre Tkatchenko. Quantum-chemical insights from deep tensor neural networks. *Nature communications*, 8(1):1–8, 2017.

[27] Dzmitry Bahdanau, Kyunghyun Cho, and Yoshua Bengio. Neural machine translation by jointly learning to align and translate. *arXiv preprint arXiv:1409.0473*, 2014.

[28] Petar Veličković, Guillem Cucurull, Arantxa Casanova, Adriana Romero, Pietro Lio, and Yoshua Bengio. Graph attention networks. *arXiv preprint arXiv:1710.10903*, 2017.

[29] Xavier Bresson and Thomas Laurent. Residual gated graph convnets. *arXiv preprint arXiv:1711.07553*, 2017.

[30] Adam Paszke, Sam Gross, Francisco Massa, Adam Lerer, James Bradbury, Gregory Chanan, Trevor Killeen, Zeming Lin, Natalia Gimelshein, Luca Antiga, et al. Pytorch: An imperative style, high-performance deep learning library. In *Advances in Neural Information Processing Systems*, pages 8024–8035, 2019.

[31] Minjie Wang, Lingfan Yu, Da Zheng, Quan Gan, Yu Gai, Zihao Ye, Mufei Li, Jinjing Zhou, Qi Huang, Chao Ma, et al. Deep graph library: Towards efficient and scalable deep learning on graphs. *arXiv preprint arXiv:1909.01315*, 2019.

[32] Jimmy Lei Ba, Jamie Ryan Kiros, and Geoffrey E Hinton. Layer normalization. *arXiv preprint arXiv:1607.06450*, 2016.

[33] Kaiming He, Xiangyu Zhang, Shaoqing Ren, and Jian Sun. Delving deep into rectifiers: Surpassing human-level performance on imagenet classification. In *Proceedings of the IEEE international conference on computer vision*, pages 1026–1034, 2015.

[34] Noel M O'Boyle, Michael Banck, Craig A James, Chris Morley, Tim Vandermeersch, and Geoffrey R Hutchison. Open babel: An open chemical toolbox. *Journal of cheminformatics*, 3(1):33, 2011.

[35] L. C. Blum and J.-L. Reymond. 970 million druglike small molecules for virtual screening in the chemical universe database GDB-13. *J. Am. Chem. Soc.*, 131:8732, 2009.

[36] M. Rupp, A. Tkatchenko, K.-R. Müller, and O. A. von Lilienfeld. Fast and accurate modeling of molecular atomization energies with machine learning. *Physical Review Letters*, 108:058301, 2012.

[37] Yang You, Jing Li, Sashank Reddi, Jonathan Hseu, Sanjiv Kumar, Srinadh Bhojanapalli, Xiaodan Song, James Demmel, and Cho-Jui Hsieh. Large batch optimization for deep learning: Training bert in 76 minutes. *arXiv preprint arXiv:1904.00962*, 1(5), 2019.

[38] Ilya Loshchilov and Frank Hutter. Fixing weight decay regularization in adam. *arXiv preprint arXiv:1711.05101*, 2017.

[39] Pavel Izmailov, Dmitrii Podoprikhin, Timur Garipov, Dmitry Vetrov, and Andrew Gordon Wilson. Averaging weights leads to wider optima and better generalization. *arXiv preprint arXiv:1803.05407*, 2018.

[40] Chi Chen, Weike Ye, Yunxing Zuo, Chen Zheng, and Shyue Ping Ong. Graph networks as a universal machine learning framework for molecules and crystals. *Chemistry of Materials*, 31(9):3564–3572, 2019.

[41] Jörg Behler. Atom-centered symmetry functions for constructing high-dimensional neural network potentials. *The Journal of Chemical Physics*, 134(7):074106, 2011.

[42] Brandon Anderson, Truong Son Hy, and Risi Kondor. Cormorant: Covariant molecular neural networks. In *Advances in Neural Information Processing Systems*, pages 14510–14519, 2019.

S14 Competition Rules

One account per participant

You cannot sign up to Kaggle from multiple accounts and therefore you cannot submit from multiple accounts.

No private sharing outside teams

Privately sharing code or data outside of teams is not permitted. It's okay to share code if made available to all participants on the forums.

Team Mergers

Team mergers are allowed and can be performed by the team leader. In order to merge, the combined team must have a total submission count less than or equal to the maximum allowed as of the merge date. The maximum allowed is the number of submissions per day multiplied by the number of days the competition has been running.

Team Limits

The maximum team size is 5.

Submission Limits

You may submit a maximum of 5 entries per day.

You may select up to 2 final submissions for judging.

Competition Timeline

Start Date: May 29, 2019

Merger Deadline: August 21, 2019

Entry Deadline: August 21, 2019

End Date (Final Submission Deadline): **August 28, 2019 11:59 PM UTC**

COMPETITION-SPECIFIC TERMS

COMPETITION TITLE: Predicting Molecular Properties

COMPETITION SPONSOR: University of Bristol

COMPETITION SPONSOR ADDRESS: University of Bristol, Beacon House, Queens Road, Bristol, BS8 1QU, United Kingdom

COMPETITION WEBSITE: https://www.kaggle.com/c/champs-scalar-coupling

TOTAL PRIZES AVAILABLE: $30,000

First Prize: $12,500

Second Prize: $7,500

Third Prize: $5,000

Fourth Prize: $3,000

Fifth Prize: $2,000

WINNER LICENSE TYPE: Open-source, MIT

Competitions are open to residents of the United States and worldwide, except that if you are a resident of Crimea, Cuba, Iran, Syria, North Korea, Sudan, or are subject to U.S. export controls or sanctions, you may not enter the Competition. Other local rules and regulations may apply to you, so please check your local laws to ensure that you are eligible to participate in skills-based competitions. The Competition Sponsor reserves the right to award alternative Prizes where needed to comply with local laws.

ENTRY IN THIS COMPETITION CONSTITUTES YOUR ACCEPTANCE OF THESE OFFICIAL COMPETITION RULES.

The Competition named above is a skills-based competition to promote and further the field of data science. You must register via the Competition Website to enter. Your competition submissions ("Submissions") must conform to the requirements stated on the Competition Website. Your Submissions will be scored based on the evaluation metric described on the Competition Website. Subject to compliance with the Competition Rules, Prizes, if any, will be awarded to participants with the best scores, based on the merits of the data science models submitted. See below for the complete Competition Rules.

A. COMPETITION-SPECIFIC RULES

In addition to the provisions of the General Competition Rules below, you understand and agree to these Competition-Specific Rules required by the Competition Sponsor:

ELIGIBILITY

The following provision supersedes General Competition Rules at Section 2.B. below: Employees, interns, contractors, officers and directors of the Competition Entities (as defined below) may enter and participate in the Competition and win prizes if they are not associated with the Chemistry and Mathematics in Phase Space (CHAMPS) Project, led by the Competition Sponsor. "Competition Entities" means the Competition Sponsor, Cardiff University, Imperial College of Science, Technology and Medicine, The University of Leeds, Kaggle Inc., and their respective parent companies, subsidiaries and affiliates. If you are such a participant from a Competition Entity, you are subject to all applicable internal policies of your employer with respect to your participation.

EXTERNAL DATA

The following provision supersedes General Competition Rule at section 7 C below: You may use data other than the Competition Data ("External Data") to develop and test your models and Submissions excluding data from quantum mechanics computations made specifically for the competition. However, you will (i) ensure the External Data is available to use by all participants of the competition for purposes of the competition at no cost to the other participants and (ii) post such access to the External Data for the participants to the official competition forum prior to the Entry Deadline.

WINNER LICENCE

Under Section 11 (Winners Obligations) of the General Competition Rules below, you hereby grant and will grant Competition Sponsor the following license(s) with respect to your Submission if you are a Competition winner:

Open Source: You hereby license and will license your winning Submission and the source code used to generate the Submission under the MIT license (see https://opensource.org/licenses/MIT).

B. GENERAL COMPETITION RULES

1. BINDING AGREEMENT.

To enter the Competition, you must agree to these Official Competition Rules, which incorporate by reference the provisions and content of the Competition Website and any Specific Competition Rules above (collectively, the "Rules"). Please read these Rules carefully before entry to ensure you understand and agree. You further agree that submission of an entry in the Competition constitutes agreement to these Rules. You may not submit an entry to the Competition and are not eligible to receive the prizes associated with this Competition ("Prizes") unless you agree to these Rules. These Rules form a binding legal agreement between you and the Competition Sponsor with respect to the Competition.

2. ELIGIBILITY.

A. To be eligible to enter the Competition, you must be: (i) a registered account holder at Kaggle.com; (ii) the older of 18 years old or the age of majority in your jurisdiction of residence (unless otherwise agreed to by Competition Sponsor and appropriate parental/guardian consents have been obtained by Competition Sponsor); (iii) not a resident of Crimea, Cuba, Iran, Syria, North Korea, or Sudan; and (iv) not a person or representative of an entity under U.S. export controls or sanctions (see https://www.treasury.gov/resource-center/sanctions/Programs/Pages/Programs.aspx).

If you are entering as a representative of a company, educational institution or other legal entity, or on behalf of your employer, these rules are binding on you, individually, and the entity you represent or are an employee. If you are acting within the scope of your employment, as an employee, contractor, or agent of another party, you warrant that such party has full knowledge of your actions and has consented thereto, including your potential receipt of a Prize. You further warrant that your actions do not violate your employer's or entity's policies and procedures.

The Competition Sponsor reserves the right to verify eligibility and to adjudicate on any dispute at any time. If you provide any false information relating to the Competition concerning your identity, residency, mailing address, telephone number, email address, ownership of right, or information required for entering the Competition, you may be immediately disqualified from the Competition.

B. Unless otherwise stated in the Specific Competition Rules above or prohibited by internal policies of the Competition Entities, employees, interns, contractors, officers and directors of Competition Entities may enter and participate in the Competition, but are not eligible to win any Prizes. "Competition Entities" means the Competition Sponsor, Kaggle Inc., and their respective parent companies, subsidiaries and affiliates. If you are such a participant from a Competition Entity, you are subject to all applicable internal policies of your employer with respect to your participation.

3. SPONSOR AND HOSTING PLATFORM.

The Competition is sponsored by Competition Sponsor named above. The Competition is hosted on behalf of Competition Sponsor by Kaggle Inc. ("Kaggle"). Kaggle is an independent contractor of Competition Sponsor, and is not a party to this or any agreement between you and Competition Sponsor. You understand that Kaggle has no responsibility with respect to selecting the potential Competition winner(s) or awarding any Prizes. Kaggle will perform certain administrative functions relating to hosting the Competition, and you agree to abide by the provisions relating to Kaggle under these Rules. As a Kaggle.com account holder and user of the Kaggle competition platform, remember you have accepted and are subject to the Kaggle Terms of Service at www.kaggle.com/terms in addition to these Rules.

4. COMPETITION PERIOD.

For the purposes of Prizes, the Competition will run from the Start Date and time to the End Date and time (such duration the “Competition Period”). The Competition Timeline is subject to change, and Competition Sponsor may introduce additional hurdle deadlines during the Competition Period. Any updated or additional deadlines will be publicized on the Competition Website. It is your responsibility to check the Competition Website regularly to stay informed of any deadline changes. *YOU ARE RESPONSIBLE FOR DETERMINING THE CORRESPONDING TIME ZONE IN YOUR LOCATION.*

5. COMPETITION ENTRY.

NO PURCHASE NECESSARY TO ENTER OR WIN. To enter the Competition, you must register on the Competition Website prior to the Entry Deadline, and follow the instructions for developing and entering your Submission through the Competition Website. Your Submissions must be made in the manner and format, and in compliance with all other requirements, stated on the Competition Website (the "Requirements"). Submissions must be received before any Submission deadlines stated on the Competition Website. Submissions not received by the stated deadlines will not be eligible to receive a Prize.

Submissions may not use or incorporate information from hand labeling or human prediction of the validation dataset or test data records.

If the Competition is a multi-stage competition with temporally separate training and/or test data, one or more valid Submissions may be required during each Competition stage in the manner described on the Competition Website in order for the Submissions to be Prize eligible.

Submissions are void if they are in whole or part illegible, incomplete, damaged, altered, counterfeit, obtained through fraud, or late. Competition Sponsor reserves the right to disqualify any entrant who does not follow these Rules, including making a Submission that does not meet the Requirements.

6. INDIVIDUALS AND TEAMS.

A. *Individual Account*. You may make Submissions only under one, unique Kaggle.com account. You will be disqualified if you make Submissions through more than one Kaggle account, or attempt to falsify an account to act as your proxy. You may submit up to the maximum number of Submissions per day as specified on the Competition Website.

B. *Teams*. If permitted under the Competition Website guidelines, multiple individuals may collaborate as a team (a "Team"); however, you may join or form only one Team. Each Team member must be a single individual with a separate Kaggle account. You must register individually for the Competition before joining a Team. You must confirm your Team membership to make it official by responding to the Team notification message sent to your Kaggle account. Team membership may not exceed the Maximum Team Size stated on the Competition Website.

C. *Team Merger*. Teams may request to merge via the Competition Website. Team mergers may be allowed provided that: (i) the combined Team does not exceed the Maximum Team Size; (ii) the number of Submissions made by the merging Teams does not exceed the number of Submissions permissible for one Team at the date of the merger request; (iii) the merger is completed before the earlier of: any merger deadline or the Competition deadline; and (iv) the proposed combined Team otherwise meets all the requirements of these Rules.

7. COMPETITION DATA.

"Competition Data" means the data or datasets available from the Competition Website for the purpose of use in the Competition, including any prototype or executable code provided on the Competition Website. The Competition Data will contain private and public test sets. Which data belongs to which set will not be made available to participants.

A. *Data Access and Use*.

Competition Use and Commercial are checked: You may access and use the Competition Data for any purpose, whether commercial or non-commercial, including for participating in the Competition and on Kaggle.com forums, and for academic research and education. The Competition Sponsor reserves the right to disqualify any participant who uses the Competition Data other than as permitted by the Competition Website and these Rules.

The Competition Data is also subject to open-source CC-BY license.

B. *Data Security*. You agree to use reasonable and suitable measures to prevent persons who have not formally agreed to these Rules from gaining access to the Competition Data. You agree not to transmit, duplicate, publish, redistribute or otherwise provide or make available the Competition Data to any party not participating in the Competition. You agree to notify Kaggle immediately upon learning of any possible unauthorized transmission of or unauthorized access to the Competition Data and agree to work with Kaggle to rectify any unauthorized transmission or access.

C. *External Data*. You may use data other than the Competition Data (“External Data”) to develop and test your models and Submissions. However, you will (i) ensure the External Data is available to use by all participants of the competition for purposes of the competition at no cost to the other participants and (ii) post such access to the External Data for the participants to the official competition forum prior to the Entry Deadline.

8. SUBMISSION CODE REQUIREMENTS.

A. *Private Code Sharing*. Unless otherwise specifically permitted under the Competition Website or Competition Specific Rules above, during the Competition Period, you are not allowed to privately share source or executable code developed in connection with or based upon the Competition Data or other source or executable code relevant to the Competition (“Competition Code”). This prohibition includes sharing Competition Code between separate Teams, unless a Team merger occurs. Any such sharing of Competition Code is a breach of these Competition Rules and may result in disqualification.

B. *Public Code Sharing*. You are permitted to publicly share Competition Code, provided that such public sharing does not violate the intellectual property rights of any third party. If you do choose to share Competition Code or other such code, you are required to share it on Kaggle.com on the discussion forum or kernels associated specifically with the Competition for the benefit of all competitors. By so sharing, you are deemed to have licensed the shared code under an Open Source Initiative-approved license (see www.opensource.org) that in no event limits commercial use of such Competition Code or model containing or depending on such Competition Code.

C. *Use of Open Source*. Unless otherwise stated in the Specific Competition Rules above, if open source code is used in the model to generate the Submission, then you must only use open source code licensed under an Open Source Initiative-approved license (see www.opensource.org) that in no event limits commercial use of such code or model containing or depending on such code.

9. DETERMINING WINNERS.

Each Submission will be scored and ranked by the evaluation metric stated on the Competition Website. During the Competition Period, the current ranking will be visible on the Competition Website's public leaderboard. The potential winner(s) are determined solely by the leaderboard ranking on the private leaderboard, subject to compliance with these Rules. The public leaderboard will be based on the public test set and the private leaderboard will be based on the private test set.

In the event of a tie, the Submission that was entered first to the Competition will be the winner. In the event a potential winner is disqualified for any reason, the Submission that received the next highest score rank will be chosen as the potential winner.

10. NOTIFICATION OF WINNERS & DISQUALIFICATION.

The potential winner(s) will be notified by email.

If a potential winner (i) does not respond to the notification attempt within one (1) week from the first notification attempt or (ii) notifies Kaggle within one week after the End Date that the potential winner does not want to be nominated as a winner or does not want to receive a Prize, then, in each case (i) and (ii) such potential winner will not receive any Prize, and an alternate potential winner will be selected from among all eligible entries received based on the Competition's judging criteria.

In case (i) and (ii) above Kaggle may disqualify the participant. However, in case (ii) above, if requested by Kaggle, such potential winner may provide code and documentation to verify the participant's compliance with these Rules. If the potential winner provides code and documentation to the satisfaction of Kaggle, the participant will not be disqualified pursuant to this paragraph.

Competition Sponsor reserves the right to disqualify any participant from the Competition if the Competition Sponsor reasonably believes that the participant has attempted to undermine the legitimate operation of the Competition by cheating, deception, or other unfair playing practices or abuses, threatens or harasses any other participants, Competition Sponsor or Kaggle.

A disqualified participant may be removed from the Competition leaderboard, at Kaggle's sole discretion. If a Participant is removed from the Competition Leaderboard, additional winning features associated with the Kaggle competition platform, for example Kaggle points or medals, may also not be awarded.

The final leaderboard list will be publicly displayed at Kaggle.com. Determinations of Competition Sponsor are final and binding.

11. WINNERS OBLIGATIONS.

As a condition to being awarded a Prize, a Prize winner must fulfill the following obligations:

(a) deliver to the Competition Sponsor the final model's software code as used to generate the winning Submission and associated documentation. The delivered software code should follow these documentation guidelines, must be capable of generating the winning Submission, and contain a description of resources required to build and/or run the executable code successfully;

(b) grant to the Competition Sponsor the license to the winning Submission stated in the Competition Specific Rules above, and represent that you have the unrestricted right to grant that license;

(c) sign and return all Prize acceptance documents as may be required by Competition Sponsor or Kaggle, including without limitation: (i) eligibility certifications; (ii) licenses, releases and other agreements required under the Rules; and (iii) U.S. tax forms (such as IRS Form W-9 if U.S. resident, IRS Form W-8BEN if foreign resident, or future equivalents).

12. PRIZES.

Prize(s) are as described on the Competition Website and are only available for winning during the time period described on the Competition Website. The odds of winning any Prize depends on the number of eligible Submissions received during the Competition Period and the skill of the participants.

All Prizes are subject to Competition Sponsor's review and verification of the participant's eligibility and compliance with these Rules, and the compliance of the winning Submissions with the Submissions Requirements. In the event that the Submission demonstrates non-compliance with these Competition Rules, Competition Sponsor may at its discretion take either of the following actions: (i) disqualify the Submission(s); or (ii) require the potential winner to remediate within one week after notice all issues identified in the Submission(s) (including, without limitation, the resolution of license conflicts, the fulfillment of all obligations required by software licenses, and the removal of any software that violates the software restrictions).

A potential winner may decline to be nominated as a Competition winner in accordance with Section 10.

Potential winners must return all required Prize acceptance documents within two (2) weeks following notification of such required documents, or such potential winner will be deemed to have forfeited the prize and another potential winner will be selected. Prize(s) will be awarded within approximately 30 days after receipt by Competition Sponsor or Kaggle of the required Prize acceptance documents. Transfer or assignment of a Prize is not allowed.

You are not eligible to receive any Prize if you do not meet the Eligibility requirements in Section 2 above.

If a Team wins a monetary Prize, the Prize money will be allocated in even shares between the eligible Team members, unless the Team unanimously opts for a different Prize split and notifies Kaggle before Prizes are issued.

13. TAXES.

ALL TAXES IMPOSED ON PRIZES ARE THE SOLE RESPONSIBILITY OF THE WINNERS. Payments to potential winners are subject to the express requirement that they submit all documentation requested by Competition Sponsor or Kaggle for compliance with applicable state, federal, local and foreign (including provincial) tax reporting and withholding requirements. Prizes will be net of any taxes that Competition Sponsor is required by law to withhold. If a potential winner fails to provide any required documentation or comply with applicable laws, the Prize may be forfeited and Competition Sponsor may select an alternative potential winner. Any winners who are U.S. residents will receive an IRS Form-1099 in the amount of their Prize.

14. GENERAL CONDITIONS.

All federal, state, provincial and local laws and regulations apply.

15. PUBLICITY.

By accepting a Prize, you agree that Competition Sponsor, Kaggle and its affiliates may use your name and likeness for advertising and promotional purposes without additional compensation, unless prohibited by law.

16. PRIVACY.

You acknowledge and agree that Competition Sponsor and Kaggle may collect, store, share and otherwise use personally identifiable information provided by you during the Kaggle account registration process and the Competition, including but not limited to, name, mailing address, phone number, and email address (“Personal Information”). Kaggle acts as an independent controller with regard to its collection, storage, sharing, and other use of this Personal Information, and will use this Personal Information in accordance with its Privacy Policy <www.kaggle.com/privacy>, including for administering the Competition. As a Kaggle.com account holder, you have the right to request access to, review, rectification, portability or deletion of any personal data held by Kaggle about you by logging into your account and/or contacting Kaggle Support at <www.kaggle.com/contact>.

17. WARRANTY, INDEMNITY AND RELEASE.

You warrant that your Submission is your own original work and, as such, you are the sole and exclusive owner and rights holder of the Submission, and you have the right to make the Submission and grant all required licenses. You agree not to make any Submission that: (i) infringes any third party proprietary rights, intellectual property rights, industrial property rights, personal or moral rights or any other rights, including without limitation, copyright, trademark, patent, trade secret, privacy, publicity or confidentiality obligations, or defames any person; or (ii) otherwise violates any applicable U.S. or foreign state or federal law.

To the maximum extent permitted by law, you indemnify and agree to keep indemnified Competition Entities at all times from and against any liability, claims, demands, losses, damages, costs and expenses resulting from any of your acts, defaults or omissions and/or a breach of any warranty set forth herein. To the maximum extent permitted by law, you agree to defend, indemnify and hold harmless the Competition Entities from and against any and all claims, actions, suits or proceedings, as well as any and all losses, liabilities, damages, costs and expenses (including reasonable attorneys fees) arising out of or accruing from: (a) your Submission or other material uploaded or otherwise provided by you that infringes any third party proprietary rights, intellectual property rights, industrial property rights, personal or moral rights or any other rights, including without limitation, copyright, trademark, patent, trade secret, privacy, publicity or confidentiality obligations, or defames any person; (b) any misrepresentation made by you in connection with the Competition; (c) any non-compliance by you with these Rules or any applicable U.S. or foreign state or federal law; (d) claims brought by persons or entities other than the parties to these Rules arising from or related to your involvement with the Competition; and (e) your acceptance,

possession, misuse or use of any Prize, or your participation in the Competition and any Competition-related activity.

You hereby release Competition Entities from any liability associated with: (a) any malfunction or other problem with the Competition Website; (b) any error in the collection, processing, or retention of any Submission; or (c) any typographical or other error in the printing, offering or announcement of any Prize or winners.

18. INTERNET.

Competition Entities are not responsible for any malfunction of the Competition Website or any late, lost, damaged, misdirected, incomplete, illegible, undeliverable, or destroyed Submissions or entry materials due to system errors, failed, incomplete or garbled computer or other telecommunication transmission malfunctions, hardware or software failures of any kind, lost or unavailable network connections, typographical or system/human errors and failures, technical malfunction(s) of any telephone network or lines, cable connections, satellite transmissions, servers or providers, or computer equipment, traffic congestion on the Internet or at the Competition Website, or any combination thereof, which may limit a participant's ability to participate.

19. RIGHT TO CANCEL, MODIFY OR DISQUALIFY.

If for any reason the Competition is not capable of running as planned, including infection by computer virus, bugs, tampering, unauthorized intervention, fraud, technical failures, or any other causes which corrupt or affect the administration, security, fairness, integrity, or proper conduct of the Competition, Competition Sponsor reserves the right to cancel, terminate, modify or suspend the Competition. Competition Sponsor further reserves the right to disqualify any participant who tampers with the submission process or any other part of the Competition or Competition Website. Any attempt by a participant to deliberately damage any website, including the Competition Website, or undermine the legitimate operation of the Competition is a violation of criminal and civil laws. Should such an attempt be made, Competition Sponsor and Kaggle each reserves the right to seek damages from any such participant to the fullest extent of the applicable law.

20. NOT AN OFFER OR CONTRACT OF EMPLOYMENT.

Under no circumstances will the entry of a Submission, the awarding of a Prize, or anything in these Rules be construed as an offer or contract of employment with Competition Sponsor or any of the Competition Entities. You acknowledge that you have submitted your Submission voluntarily and not in confidence or in trust. You acknowledge that no confidential, fiduciary, agency, employment or other similar relationship is created between you and Competition Sponsor or any of the Competition Entities by your acceptance of these Rules or your entry of your Submission.

21. GOVERNING LAW.

Unless otherwise provided in the Competition Specific Rules above, all claims arising out of or relating to these Rules will be governed by California law, excluding its conflict of laws rules, and will be litigated exclusively in the Federal or State courts of Santa Clara County,

California, USA. The parties consent to personal jurisdiction in those courts. If any provision of these Rules is held to be invalid or unenforceable, all remaining provisions of the Rules will remain in full force and effect.

S15 Competition description

Think you can use your data science smarts to make big predictions at a molecular level?

This challenge aims to predict interactions between atoms. Imaging technologies like MRI enable us to see and understand the molecular composition of tissues. Nuclear Magnetic Resonance (NMR) is a closely related technology which uses the same principles to understand the structure and dynamics of proteins and molecules.

Researchers around the world conduct NMR experiments to further understanding of the structure and dynamics of molecules, across areas like environmental science, pharmaceutical science, and materials science.

This competition is hosted by members of the CHemistry and Mathematics in Phase Space (CHAMPS) at the University of Bristol, Cardiff University, Imperial College and the University of Leeds. Winning teams will have an opportunity to partner with this multi-university research program on an academic publication

Your Challenge

In this competition, you will develop an algorithm that can predict the magnetic interaction between two atoms in a molecule (i.e., the scalar coupling constant).

Once the competition finishes, CHAMPS would like to invite the top teams to present their work, discuss the details of their models, and work with them to write a joint research publication which discusses an open-source implementation of the solution.

About Scalar Coupling

Using NMR to gain insight into a molecule's structure and dynamics depends on the ability to accurately predict so-called "scalar couplings". These are effectively the magnetic interactions between a pair of atoms. The strength of this magnetic interaction depends on intervening electrons and chemical bonds that make up a molecule's three-dimensional structure.

Using state-of-the-art methods from quantum mechanics, it is possible to accurately calculate scalar coupling constants given only a 3D molecular structure as input. However, these quantum mechanics calculations are extremely expensive (days or weeks per molecule), and therefore have limited applicability in day-to-day workflows.

A fast and reliable method to predict these interactions will allow medicinal chemists to gain structural insights faster and cheaper, enabling scientists to understand how the 3D chemical structure of a molecule affects its properties and behavior.

Ultimately, such tools will enable researchers to make progress in a range of important problems, like designing molecules to carry out specific cellular tasks, or designing better drug molecules to fight disease.

Join the CHAMPS Scalar Coupling challenge to apply predictive analytics to chemistry and chemical biology.
