# Supplementary material for: A community-powered search of machine learning strategy space to find NMR property prediction models
Source: PLoS One. 2021 Jul 20;16(7):e0253612. doi: 10.1371/journal.pone.0253612 (PMC8291653; doi:10.1371/journal.pone.0253612)
Supplement: Attachment — Submitted filename: Response to Reviewers.pdf [file pone.0253612.s002.pdf]

Journal Requirements:

Please review your reference list to ensure that it is complete and correct. If you have cited papers that have been retracted, please include the rationale for doing so in the manuscript text, or remove these references and replace them with relevant current references. Any changes to the reference list should be mentioned in the rebuttal letter that accompanies your revised manuscript. If you need to cite a retracted article, indicate the article's retracted status in the References list and also include a citation and full reference for the retraction notice.

We fixed a few instances of duplicate citations and to our knowledge the references are complete and correct. The title of reference 28 includes a link that is no longer active, but the reference is still correct.

1. Please ensure that your manuscript meets PLOS ONE's style requirements, including those for file naming. The PLOS ONE style templates can be found at

https://journals.plos.org/plosone/s/file?id=wjVg/PLOSOne_formatting_sample_main_body.pdf and

https://journals.plos.org/plosone/s/file?id=ba62/PLOSOne_formatting_sample_title_authors_affiliations.pdf

We have made slight formatting changes to follow the guidelines better. We have only included the supporting information (SI) as a single pdf, as we don't directly reference any figures, and due to the main document being written in Microsoft Word and the SI being written in latex.

2. Please include information about the competition, participant recruitment and participant consent in the Methods section. Please include details regarding participant consent to collect personal data, including email addresses, names, or phone numbers. In the Methods section, please ensure that you have specified how consent was obtained and how the study met relevant personal data and privacy laws. If data were collected anonymously, please include this information.

Moved the content from what was previously section S2 which contained this information to the main text as section 3.3. The competition description and rules were added to the SI.

3. Thank you for stating the following in the Competing Interests section:

"I have read the journal's policy and the authors of this manuscript have the following competing interests: This competition was made possible through financial support from Kaggle, where AH and WR are employees."

We note that one or more of the authors are employed by a commercial company: BNP Paribas Cardif, Fyusion Inc., Kaggle, Google Inc., & Ebay Korea, MINDS AND COMPANY, Totient Inc.

3.1. Please provide an amended Funding Statement declaring this commercial affiliation, as well as a statement regarding the Role of Funders in your study. If the funding organization did not play a role in the study design, data collection and analysis, decision to publish, or preparation of the manuscript and only provided financial support in the form of authors' salaries and/or research materials, please review your statements relating to the author contributions, and ensure you have specifically and accurately indicated the role(s) that these authors had in your study. You can update author roles in the Author Contributions section of the online submission form.

Please also include the following statement within your amended Funding Statement.

“The funder provided support in the form of salaries for authors [insert relevant initials], but did not have any additional role in the study design, data collection and analysis, decision to publish, or preparation of the manuscript. The specific roles of these authors are articulated in the ‘author contributions’ section.”

If your commercial affiliation did play a role in your study, please state and explain this role within your updated Funding Statement.

3.2. Please also provide an updated Competing Interests Statement declaring this commercial affiliation along with any other relevant declarations relating to employment, consultancy, patents, products in development, or marketed products, etc.

Within your Competing Interests Statement, please confirm that this commercial affiliation does not alter your adherence to all PLOS ONE policies on sharing data and materials by including the following statement: "This does not alter our adherence to PLOS ONE policies on sharing data and materials.” (as detailed online in our guide for authors http://journals.plos.org/plosone/s/competing-interests) . If this adherence statement is not accurate and there are restrictions on sharing of data and/or materials, please state these. Please note that we cannot proceed with consideration of your article until this information has been declared.

Please include both an updated Funding Statement and Competing Interests Statement in your cover letter. We will change the online submission form on your behalf.

Please know it is PLOS ONE policy for corresponding authors to declare, on behalf of all authors, all potential competing interests for the purposes of transparency. PLOS defines a competing interest as anything that interferes with, or could reasonably be perceived as

interfering with, the full and objective presentation, peer review, editorial decision-making, or publication of research or non-research articles submitted to one of the journals. Competing interests can be financial or non-financial, professional, or personal. Competing interests can arise in relationship to an organization or another person. Please follow this link to our website for more details on competing interests:
http://journals.plos.org/plosone/s/competing-interests

These companies (other than Kaggle as was already mentioned) did not provide any funding towards the competition and has no financial interest in the manuscript.

4. We note that Figure 1 in your submission contain map images which may be copyrighted. All PLOS content is published under the Creative Commons Attribution License (CC BY 4.0), which means that the manuscript, images, and Supporting Information files will be freely available online, and any third party is permitted to access, download, copy, distribute, and use these materials in any way, even commercially, with proper attribution. For these reasons, we cannot publish previously copyrighted maps or satellite images created using proprietary data, such as Google software (Google Maps, Street View, and Earth). For more information, see our copyright guidelines:
http://journals.plos.org/plosone/s/licenses-and-copyright.

We require you to either (1) present written permission from the copyright holder to publish these figures specifically under the CC BY 4.0 license, or (2) remove the figures from your submission:

4.1. You may seek permission from the original copyright holder of Figure 1 to publish the content specifically under the CC BY 4.0 license.

We recommend that you contact the original copyright holder with the Content Permission Form (http://journals.plos.org/plosone/s/file?id=7c09/content-permission-form.pdf) and the following text:

“I request permission for the open-access journal PLOS ONE to publish XXX under the Creative Commons Attribution License (CCAL) CC BY 4.0 (http://creativecommons.org/licenses/by/4.0/). Please be aware that this license allows unrestricted use and distribution, even commercially, by third parties. Please reply and provide explicit written permission to publish XXX under a CC BY license and complete the attached form.”

Please upload the completed Content Permission Form or other proof of granted permissions as an "Other" file with your submission.

In the figure caption of the copyrighted figure, please include the following text: “Reprinted from [ref] under a CC BY license, with permission from [name of publisher], original copyright [original copyright year].”

4.2. If you are unable to obtain permission from the original copyright holder to publish these figures under the CC BY 4.0 license or if the copyright holder’s requirements are incompatible with the CC BY 4.0 license, please either i) remove the figure or ii) supply a replacement figure that complies with the CC BY 4.0 license. Please check copyright information on all replacement figures and update the figure caption with source information. If applicable, please specify in the figure caption text when a figure is similar but not identical to the original image and is therefore for illustrative purposes only.

The following resources for replacing copyrighted map figures may be helpful:

USGS National Map Viewer (public domain): http://viewer.nationalmap.gov/viewer/

The Gateway to Astronaut Photography of Earth (public domain): http://eol.jsc.nasa.gov/sseop/clickmap/

Maps at the CIA (public domain): https://www.cia.gov/library/publications/the-world-factbook/index.html and https://www.cia.gov/library/publications/cia-maps-publications/index.html

NASA Earth Observatory (public domain): http://earthobservatory.nasa.gov/

Landsat: http://landsat.visibleearth.nasa.gov/

USGS EROS (Earth Resources Observatory and Science (EROS) Center) (public domain): http://eros.usgs.gov/#

Natural Earth (public domain): http://www.naturalearthdata.com/

The image in Fig 1 was generated by the authors using a Google Sheets GeoChart. Therefore the copyright is owned by the authors, and there should be no problem to publish under CC-BY

5. One of the noted authors is a group or consortium [Kaggle participants]. In addition to naming the author group, please list the individual authors and affiliations within this group in the acknowledgments section of your manuscript. Please also indicate clearly a lead author for this group along with a contact email address.

We clarified the authorships slightly in the main text formatting. ‘Kaggle participants’ is not a consortium or group, but is giving credit to all private participants in a Kaggle-run competition that is the basis for this manuscript. The descriptor is used as a grouping affiliation for those authors which do not wish to use their employment details as a separate affiliation. As such, they had no lead author or organised structure and each contributed to the data which is analysed in the paper.

6. Please include captions for your Supporting Information files at the end of your manuscript, and update any in-text citations to match accordingly. Please see our Supporting Information guidelines for more information:
http://journals.plos.org/plosone/s/supporting-information.

SI was written in Latex, while the main text was written in Microsoft Word. Together with the specific information and layout of the SI, we added a ‘Supporting information’ section to the manuscript with descriptions of the SI sections, rather than individual captions.

Reviewer 1:

While I understand why the comparison is with computed structures and QM calculations it is a pity that experimental work can not be included and a proper assessment of the importance of experimental uncertainty included in the study.

We feel like any discussion about practical use and possible issues with applying parameters obtained by machine learning trained on quantum mechanics data to experimental data is beyond the scope of the paper. Especially as this was not the task presented to the participants. This work is partially about exploring crowd-sourcing science, but also partly an extension of our previous work named IMPRESSION (ref 24), wherein we believe we explored this more practical question in detail.

Specific comments
The scoring function given in equation (1) is fine and justified but then I was confused to see a discussion of the geometric mean (exponential of the score??) a bit further down in the paper.

We have expanded slightly on the wording.
Equation (1) put into words is the natural logarithm of the geometric mean of the mean absolute errors for each type of coupling. This geometric mean has often been used as a single error metric for multiple different parameters on different scales of size in e.g. comparing DFT performance in NMR. Taking the log of this was practical for the competition, as we avoided different submissions differing only on the 5th decimal.
For the sake of discussion we wanted to quantify that the mean error was improved by an order of magnitude. We agree that this shift can indeed be confusing, but we nevertheless find that pointing out the connection to the geometric mean could still be useful for readers familiar with this.

The way in which remodels converged over the time of the competition is interesting but I don't think worth fitting the exponentials and making quite such a fuss about as the paper is more about the models not human behaviour (though there is certainly a paper in that as well). So I suggest that either less or more on this is discussed.

In setting up and running a crowd-sourced science competition such as this, one critical question which emerges is: "for how long should we run the competition?" We take the referee's point that our analysis here could be more in-depth; however, we believe that these results are important to include for a few reasons:

(1) They provide insight into the speed with which our best scores were matched by the community
(2) They highlight the dramatic deceleration in scores vs. time, which may provide useful insight for others who are organizing similar competitions in different scientific domains
(3) They are qualitatively aligned with the results highlighted by detailed behavioural studies such as those published in *PNAS* **112**, 14569 (2015), a point which is discussed near the end of the paper

The use of the meta ensemble is good but the whole discussion of the range of ranked members of the ensemble and the weights etc come across as rather confused. I am sure this can be simplified and made much clearer. The point that different models while displaying different scores are sensitive to different cases is interesting and very well worth exploring.

Without more specific recommendations, we are unclear exactly what the referee means. From our perspective, we believe that the main points of the discussion are clear:

(1) The optimized meta-ensemble (ME) model was 7-19x more accurate than our previously published model
(2) The optimized ME model had six constituents with weights *wi* > 0.02
(3) Within the optimized ME, no particular model is dominant
(4) Of the six models in the optimized ME, one (#12) falls outside the top 5, and in fact it has a 0.149 contribution which is larger than two of the top-5 prize winning models.
(5) Model #12 has a large contribution to the ME owing to its small correlation with the top-5 models. This is a consequence of the fact that it adopts a rather rigorous physics approach compared to other submissions

The tone of the discussion comes over and rather dismissive of the physics inspired models, perhaps because they are better. I also suggest the use of physics is not necessarily the best way to describe something that is just as much chemistry, even if it is in common use.

Here we are unclear exactly what the referee means. In fact, we believe that the tone of our discussion is actually very complementary of physics-based models! For example, we highlight that the physics inspired models (e.g, from team 12) have 100x fewer parameters compared to competitors, and also preserve several important symmetries that other models do not! We also highlight the fact that the physics approach of team 12 in fact played a more important role in the optimized meta ensemble model than some of the top-5 prize winning teams! Taken together, we believe that these comments are actually powerful arguments in

favor of physics approaches compared to more naive approaches which do not implement domain specific knowledge.

Reviewer 2:

As a final comment I admit that as a chemist involved in computational model I would have appreciated a more chemical discussion about the type of errors encountered in relation to the molecular species or conformations.

We agree that this could be interesting, and if the focus of the manuscript was a new computational method, we would be happy to include this. However, since the focus is the competition itself and the results thereof, and that the work will be of interest to non-chemists, we find that this is beyond the scope of the manuscript.

A minor detail, but in the document submitted the correlation matrix b) is useless as numbering cannot be read.

We believe that this is due to some unfortunate compression in the pdf conversion by the online submission tool. This should not affect the image quality of the final publication. A raw image of the correlation matrix in question can be found here: https://github.com/larsbratholm/champs_kaggle/blob/master/analysis/output/correlation_matrix_clustering_50.png
